# Early Metabolomic Changes During Extracorporeal Membrane Oxygenation Are Associated with Subsequent Acute Brain Injury

**DOI:** 10.3390/cells15171593

**Published:** 2026-09-01

**Authors:** Bosco Seong Kyu Yang, Yaman Ahmad, Hua Chen, Shivalika Khanduja, Zoe Soulé, Rene Leal, Huimahn A. Choi, Louise D. McCullough, Kha Dinh, Bindu Akkanti, Glenn Whitman, Sung-Min Cho, Aaron M. Gusdon

**Affiliations:** 1 Division of Neurocritical Care, Department of Neurosurgery, McGovern School of Medicine, University of Texas Health Science Center, Houston, TX 77030, USA; seong.kyu.yang@uth.tmc.edu (B.S.K.Y.); hua.chen@uth.tmc.edu (H.C.); rene.leal@uth.tmc.edu (R.L.); huimahn.a.choi@uth.tmc.edu (H.A.C.); 2 Department of Anesthesiology and Critical Care Medicine, Johns Hopkins University School of Medicine, Baltimore, MD 21287, USA; yahmad5@jh.edu (Y.A.); skhandu2@jhmi.edu (S.K.); zsoule1@jhu.edu (Z.S.); 3 Department of Neurology, McGovern School of Medicine, University of Texas Health Science Center, Houston, TX 77030, USA; louise.d.mccullough@uth.tmc.edu; 4 Divisions of Pulmonary, Critical Care, and Sleep Medicine, McGovern Medical School, the University of Texas Health Science Center at Houston, Houston, TX 77030, USA; kha.dinh@uth.tmc.edu (K.D.); bindu.h.akkanti@uth.tmc.edu (B.A.); 5 Department of Cardiothoracic Surgery, Johns Hopkins University School of Medicine, Baltimore, MD 21287, USA; gwhitman@jhmi.edu

**Keywords:** acute brain injury, metabolomics, extracorporeal membrane oxygenation

## Abstract

**Background**: Acute brain injury (ABI) is a frequent complication of extracorporeal membrane oxygenation (ECMO), but early detection is limited by sedation, imaging constraints, and low sensitivity of conventional neuroimaging. We hypothesized that plasma metabolomics could identify ECMO-mode-specific metabolic shifts and biomarkers preceding ABI. **Methods**: Untargeted plasma metabolomics was performed in 70 participants across two centers: 30 healthy controls, 17 critically ill controls, and 23 ECMO patients [14 venovenous (VV) and 9 venoarterial (VA)]. Plasma was collected within 24 h and 7 days after cannulation. Fold-change analyses and partial least squares discriminant analysis were used to define metabolic differences and identify metabolites associated with subsequent ABI. **Results**: ABI occurred in seven ECMO patients, including five venoarterial and two venovenous ECMO patients. ECMO support was associated with broad alterations in circulating lipid metabolism, including changes in sphingomyelins, lysophospholipids, and monoacylglycerols. PLS-DA demonstrated metabolomic separation between ECMO patients and controls. Among ECMO patients, three structurally related glycerophospholipids—GPI (18:0/18:2), GPC (16:0/18:2), and GPE (16:0/18:2)—were significantly decreased before ABI diagnosis. ABI was also associated with broader reductions in phosphatidylethanolamines, phosphatidylinositols, lysophospholipids, and polyunsaturated fatty acids. **Conclusions**: Early reductions in membrane-associated phospholipids were associated with subsequent ABI during ECMO support, suggesting that alterations in circulating lipid homeostasis may identify neurological vulnerability before clinical or radiographic recognition of injury. Plasma metabolomics may provide a complementary approach for early neurological risk stratification and support future biomarker development.

## 1. Introduction

Extracorporeal membrane oxygenation (ECMO) is a rescue therapy for subjects with refractory cardiac or respiratory failure. However, this lifesaving intervention entails a substantial risk of acute brain injury (ABI), including ischemic stroke, intracranial hemorrhage, hypoxic-ischemic brain injury, and brain death [1]. Approximately 15% of adult ECMO subjects experience a significant neurological complication, with the incidence rising to 30% in subjects with extracorporeal cardiopulmonary resuscitation [1,2]. However, an autopsy study on ECMO subjects suggests that this estimated incidence is potentially underestimated and could be as high as 68% [3]. This risk is especially pronounced in venoarterial ECMO (VA-ECMO) compared to venovenous ECMO (VV-ECMO) [4]. ABI occurrence during ECMO doubles the risk of mortality and is associated with poor cognitive function [5,6]. Given the profound impact of ABI on mortality and long-term neurological outcomes, strategies for early detection and prevention are urgently needed.

Early detection of ABI remains challenging in ECMO patients. Sedation and neuromuscular blockade often preclude meaningful neurological examination, removing one of the most fundamental tools for early recognition. Computed tomography (CT) of the brain is insensitive to early ischemia, while conventional magnetic resonance imaging (MRI) is largely incompatible with ECMO support due to equipment constraints and safety considerations [7,8]. Although emerging imaging modalities have been explored, ABI is typically identified only after substantial and often irreversible damage has occurred [9]. These limitations highlight the need for real-time, physiology-informed biomarkers capable of the early detection of evolving brain injury before irreversible damage occurs.

The pathophysiology of ECMO-related ABI is multifactorial, involving cerebral hypoperfusion, reperfusion injury, coagulopathy, inflammation, and device-related circulatory disruption [10,11,12,13]. Because these processes dynamically alter systemic metabolism, circulating plasma metabolites may provide early insight into the biological events that precede clinical or radiographic evidence of ABI. Metabolomics offers a powerful, systems-level approach to biomarker discovery. By simultaneously quantifying hundreds of small molecules (including lipids, amino acids, and carbohydrates), metabolomics captures the integrated downstream products of genomic, transcriptomic, proteomic, and environmental perturbations [14,15]. In contrast to upstream omics, the metabolome reflects rapid real-time physiological changes, is highly responsive to injury and stress, and directly mirrors cellular energy states, mitochondrial dysfunction, membrane breakdown, oxidative stress, and neuroinflammatory signaling, all central features of ABI pathogenesis [16,17,18]. Several key metabolite classes also play essential roles in blood–brain barrier integrity, a structure frequently disrupted during ABI [16,19]. Importantly, circulating metabolomic signatures have been shown to track the severity and evolution of traumatic brain injury, subarachnoid hemorrhage, and ischemic stroke, supporting their potential as noninvasive biomarkers of neurological injury [18,20,21,22,23,24].

In the context of ECMO, where traditional neurological assessment is limited and early injury may be clinically silent, metabolomics may offer a complementary approach to neurological risk stratification. By identifying molecular signatures that precede overt neurological deterioration, metabolomic profiling could enable earlier detection and risk assessment. Established blood-based neurological biomarkers such as GFAP, UCH-L1, NSE, and S100B primarily reflect neuronal or astroglial cellular injury. In contrast, metabolomic profiling simultaneously captures changes in membrane remodeling, energy metabolism, oxidative stress, inflammation, and endothelial function. Because these metabolic disturbances may occur during evolving physiological stress before overt structural injury is recognized, metabolomics may identify a broader state of neurological vulnerability rather than relying on a single marker of cellular injury. Whether metabolomic signatures provide additive or earlier information than established protein biomarkers requires direct comparison in future studies.

Here, we performed untargeted plasma metabolomic profiling in VA- and VV-ECMO patients with two complementary objectives. First, we sought to define ECMO mode-specific metabolic signatures and identify circulating metabolites present early after cannulation that could serve as candidate biomarkers of subsequent ABI. Second, we sought to determine which biological pathways were represented by these metabolomic changes, thereby generating mechanistic hypotheses regarding the processes that may contribute to neurological vulnerability during ECMO support. Specifically, we aimed to: (1) characterize metabolic differences associated with VA- and VV-ECMO, (2) identify early metabolomic features associated with subsequent ABI, and (3) evaluate the lipid and metabolic pathways represented by these features as potential contributors to ECMO-associated brain injury and targets for future investigation.

## 2. Materials and Methods

### 2.1. Study Population and Sample Collection

This study included subjects from four clinical groups: healthy controls, critically ill patients without neurological diagnoses (sick controls), and patients receiving ECMO support—further subdivided as veno-venous (VV) ECMO or veno-arterial (VA) ECMO. Patients were prospectively enrolled into biorepositories at two tertiary care centers: The University of Texas Health Science Center (UTHealth) and the Johns Hopkins Hospital (JHH). Patients with a pre-existing neurological disorder were excluded, including those with chronic neurodegenerative disease, to minimize potential confounding of the circulating metabolomic profile by underlying neurological pathology and to ensure that neurological outcomes represented newly acquired ABI during ECMO support. Critically ill controls were similarly selected from patients without known neurological diagnoses.

Serial blood samples were collected at two time points: within 24 h of ECMO initiation (T1) and at 7 days after cannulation (T2). At T1, ECMO patients were critically ill and undergoing procedures associated with cannulation and early stabilization and had not yet been receiving nutritional support at the time of sample collection; therefore, these samples were obtained while patients were effectively fasting/NPO. Nutritional exposure was more heterogeneous at T2, as some patients had initiated enteral or parenteral nutrition by this time point. Blood samples were processed promptly after collection by centrifugation at 1000× *g* for 10 min to prepare platelet-poor plasma and were immediately frozen at −80 °C until metabolomic analysis. All participants or their legally authorized representatives provided informed consent, and the study was approved by the Institutional Review Boards at participating centers (UTHSC IRB: HSC-MS-23-0289).

Clinical data were prospectively collected for all subjects, including demographics, clinical diagnoses, and laboratory parameters. For ECMO subjects, additional variables included indications for ECMO cannulation, central or peripheral cannulation, days on ECMO support, and pre- and post-ECMO arterial blood gas parameters (pH, bicarbonate, PaO_2_, PaCO_2_, lactate, SaO_2_).

ABI included ischemic stroke, hypoxic-ischemic brain injury (HIBI), brain death, intraparenchymal hemorrhage (IPH), subarachnoid hemorrhage (SAH), and subdural hematoma (SDH). ABI was adjudicated based on clinical documentation and imaging findings. All subjects with ABI had computed tomography (CT) images of the brain available. In addition, two subjects with ABI had ultra-low magnetic field portable MRI imaging available.

### 2.2. Metabolomic Profiling

Plasma samples collected at both participating institutions were processed using a standardized protocol, stored at −80 °C, transferred to UTHealth, and consolidated before metabolomic analysis. Samples from both centers were submitted together for untargeted metabolomic profiling and were not analyzed as separate institution-specific analytical batches. Plasma samples were analyzed using an untargeted metabolomics platform (Metabolon, Morrisville, NC, USA) [25,26,27]. The platform combines ultra-high-performance liquid chromatography–tandem mass spectrometry (UPLC-MS/MS) in positive- and negative-ion modes. Metabolite identities were confirmed by comparison with a standard reference library. Raw peak areas were normalized to internal standards, scaled according to the platform’s standard analytical workflow, and log-transformed for statistical analysis. Because the collection site was not confounded with a separate analytical run, no additional site-based batch correction was performed.

A total of 1013 metabolites were detected across the samples. After removing uncharacterized metabolites and metabolites with >5% missing rates, 842 metabolites were included in the final analysis.

### 2.3. Differential Expression Analysis

Pairwise comparisons between clinical groups were performed using the Wilcoxon rank-sum test followed by false-discovery-rate correction using the Benjamini–Hochberg method. For univariate feature prioritization, volcano-plot visualization, and pathway-level summaries, metabolites with a fold change > 2 or <0.5 and an FDR-adjusted *p*-value < 0.05 were classified as prominently altered. These fold-change thresholds were selected a priori to prioritize metabolites with larger effect sizes in this exploratory, high-dimensional dataset and were not intended to indicate that metabolites with smaller changes lacked biological relevance. All eligible metabolites were retained in the multivariate PLS-DA analyses regardless of fold change.

### 2.4. Supervised Multivariate Analysis (PLS-DA)

To evaluate whether metabolomic profiles could discriminate between predefined clinical groups, partial least squares discriminant analysis (PLS-DA) was performed using the mixOmics package in R (R version 4.3.1). Prior to modeling, metabolite intensities were log-transformed and mean-centered with unit variance scaling to ensure comparability across features. PLS-DA models were trained using a supervised multivariate framework that projects high-dimensional metabolomic data into a lower-dimensional latent space while maximizing covariance between metabolite features and group membership.

Optimally discriminating models were found using five-fold cross-validation and using the 1-standard-error rule. Classification performance was summarized using overall (OER) and balanced error rates (BER). Variable importance in projection (VIP) scores were calculated for each latent component to rank metabolites contributing most to group separation. Metabolites with higher VIP scores were interpreted as having greater influence on class separation in the multivariate model.

### 2.5. Downstream Analysis

Significant metabolites were grouped according to the macromolecular categories (Super Pathway) to which they belong and further classified depending on structurally and functionally distinct subcategories (Sub Pathway). The number of increased (log2FC > 1) and decreased (log2FC < −1) metabolites that show a significant difference between comparison groups (q < 0.05 or *p* < 0.05) in each category was counted. For each category, the proportion of altered metabolites was calculated as the count divided by the total number of significant metabolites within its immediate encompassing category.

For a selected set of metabolites with the most significant alterations between ECMO subjects with and without ABI, temporal trends were evaluated. For each selected metabolite, expression values were plotted across ABI status and time points (T1 and T2)—for temporal trends, relative values are plotted for ease of understanding. Pairwise statistical comparisons were performed using two-sided Wilcoxon rank-sum tests to assess within-group temporal changes and between-group differences at each time point. Except for the temporal trend analysis, all other analyses focused on metabolomic profiles at T1.

### 2.6. Demographics Analysis

Demographic and clinical characteristics were summarized using medians (interquartile range) or means (SD) as appropriate for continuous variables and proportions for categorical variables. Between-group comparisons were made using the Wilcoxon rank-sum test, Chi-square test, Fisher’s exact test, and Kruskal–Wallis test as appropriate. All statistical analyses were performed using R version 4.3.1.

## 3. Results

### 3.1. Patient Characteristics

A total of 70 subjects were included in this study: 30 healthy controls, 17 sick controls, nine VA-ECMO subjects, and 14 VV-ECMO subjects (Table 1). Across groups, age, race, and ethnicity distributions differed significantly, whereas sex distribution did not. Healthy and sick controls were age-matched (mean ± SD: 59.5 ± 8.8 vs. 57.9 ± 17.0 years, *p* = 0.73). In contrast, the ECMO cohort overall had a mean age of 50.0 ± 17.5 years, but, within the ECMO cohort, VA-ECMO subjects were significantly older (67.4 ± 11.9 years) than VV-ECMO subjects (38.7 ± 9.1 years; *p* < 0.001). Sex distribution was statistically identical in all subject groups. Among sick controls, the most common admission diagnoses were COPD/asthma exacerbation (47%), pneumonia (35%), sepsis (23%), and congestive heart failure (23%).

Within the ECMO subgroup, there were multiple important differences between VA- and VV-ECMO (Table 2). Most VV-ECMO patients (92.9%) required ECMO for COVID-19-associated ARDS, whereas VA-ECMO indications included cardiogenic shock (n = 5), post-cardiotomy shock (n = 3), and ARDS (n = 1). VA- and VV-ECMO subjects differed across multiple physiological parameters. VV-ECMO subjects required significantly longer ECMO support compared to VA-ECMO subjects (35.9 ± 30.0 vs. 11.3 ± 11.7 days; *p* = 0.002).

Significant differences were observed in arterial blood gas profiles. Prior to cannulation, VA-ECMO subjects had higher pH (7.4 ± 0.1 vs. 7.2 ± 0.1; *p* = 0.02), lower PaCO_2_ (32.9 ± 8.3 vs. 76.9 ± 31.0 mmHg; *p* < 0.001), and higher PaO_2_ (179.8 ± 86.5 vs. 66.4 ± 22.2 mmHg; *p* < 0.001) than VV-ECMO subjects. After cannulation, VA-ECMO subjects maintained higher oxygenation and lower carbon dioxide tension (PaO_2_ 188.3 ± 75.7 vs. 69.9 ± 20.9 mmHg; PaCO_2_ 30.9 ± 9.1 vs. 58.6 ± 9.4 mmHg; both *p* < 0.001), with correspondingly higher SaO_2_ (99.0 ± 0.8 vs. 89.5 ± 9.3%; *p* < 0.001). Pre-cannulation lactate levels did not differ significantly between groups (pre-ECMO 6.9 ± 6.6 vs. 4.0 ± 5.0 mmol/L; *p* = 0.43).

ABI occurred during the course of ECMO support in seven subjects (Table 2). ABI was more common among VA-ECMO subjects (n = 5, 55.6%) than VV-ECMO subjects (n = 2, 14.3%). Among the VA-ECMO group, four subjects were diagnosed with IS and one was diagnosed with HIBI. Conversely, in the VV-ECMO group, one case each of IPH and SAH was diagnosed.

### 3.2. ECMO vs. Controls

#### 3.2.1. VA-ECMO vs. Controls

**Figure 1:**
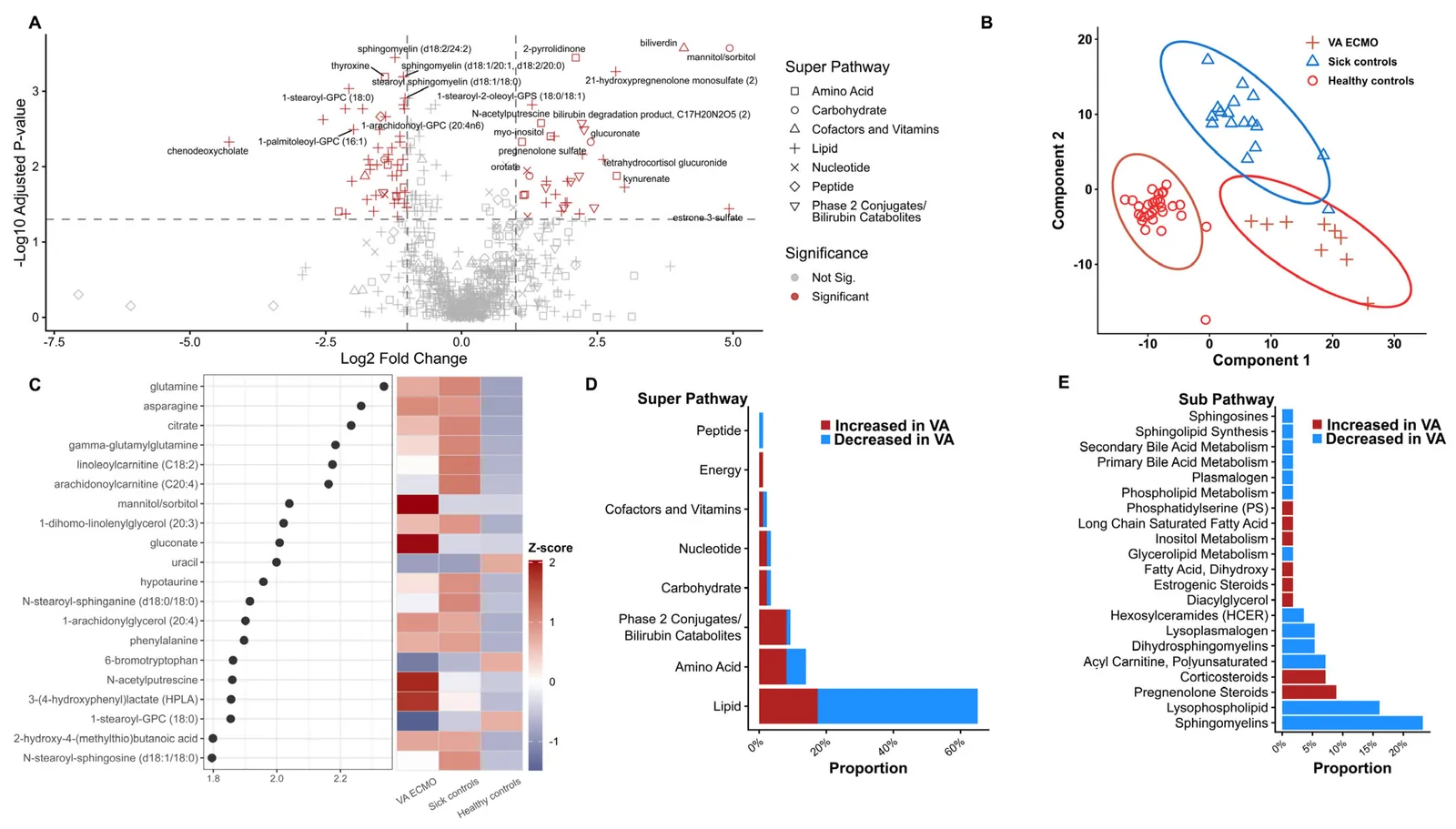
Differential metabolomic signatures in VA-ECMO subjects compared to sick and healthy controls. (**A**) Volcano plot showing differential metabolite expression between VA-ECMO and control groups. Each point represents an individual metabolite, colored by statistical significance (adjusted *p* < 0.05, |log2 fold change| > 1) and shaped by Super Pathway classification. Lipid metabolites dominate the significant changes, with several sphingomyelin and lysophospholipid species markedly decreased. (**B**) Partial Least Squares Discriminant Analysis (PLS-DA) illustrating a clear separation between VA-ECMO, sick controls, and healthy controls, suggesting distinct metabolic profiles across groups. (**C**) Variable importance plot showing glutamine and asparagine to be the most important in differentiating the three groups. (**D**) Classes of significantly altered metabolites. Lipid metabolites comprised the majority of altered metabolites with most altered lipid metabolites being decreased among VA-ECMO subjects. (**E**) Categories of significantly altered lipid metabolites demonstrating decreases in sphingomyelin and lysophospholipids among VA-ECMO subjects.

Significantly increased (FC > 2 and q < 0.05) or decreased (FC < 0.5 and q < 0.05) metabolites and their pathways are shown as a volcano plot (Figure 1A). Of 842 metabolites analyzed, 94 (11.2%) were significantly altered in VA-ECMO subjects compared with sick controls (Supplementary Table S1). The metabolites significantly decreased in VA-ECMO subjects included lipids [sphingomyelins (SM), lysophospholipids (LPL), lysoplasmalogens, and acylcarnitines], and amino acid derivatives (Leucine/isoleucine/valine metabolic products and tyrosine metabolic products), while increased metabolites included hemoglobin/porphyrin metabolic products, carbohydrate derivatives (Fructose/mannose/galactose metabolic products), and some lipids involved in steroid hormone synthesis (pregnenolone steroids, corticosteroids).

When compared to healthy controls, considerably more (283, 33.6%) metabolites were significantly altered (Supplementary Table S2). Significantly decreased metabolites among VA-ECMO subjects included a similar group of lipids [SMs, LPLs, phosphatidylcholines (PC), and plasmalogens] as well as cofactors/vitamins (thyroxine, vitamin A metabolic products) (Supplementary Figure S1A). Significantly increased metabolites included lipids [monoacylglycerols (MAG)], amino acid derivatives (polyamine, alanine/aspartate, histidine, tyrosine, glutathione, glutamate, methionine/cysteine/SAM metabolic products), nucleotides (purine/pyrimidine metabolism), and carbohydrate derivatives (fructose/mannose/galactose metabolic products).

PLS-DA readily separated the three groups based on their metabolomes. The best model required five components for group discrimination. The proportion of variance explained by the metabolomic data across the five components were 15.3%, 7.1%, 9.5%, 4.4%, and 4.3%, respectively. Classification performance was robust, with a minimum cross-validated error rate of 0.093 and balanced error rate of 0.120, indicating stable separation of VA-ECMO from both control groups (Figure 1B). The most influential metabolites by VIP scores included glutamine, asparagine, citrate, gamma-glutamylglutamine, linoleoylcarnitine (18:2), and arachidonoylcarnitine (20:4) (Figure 1C).

Across both comparisons, lipid metabolites were the most commonly altered. When VA-ECMO subjects were compared with sick controls, 56 of the 94 significantly altered metabolites (59.6%) were lipids (median log2FC = −0.98) (Figure 1D). Multiple lipid subpathways showed significant changes. Categories of lipid metabolites that were decreased among VA-ECMO subjects included SMs (13 metabolites, median log2FC = −1.02), LPLs (9 metabolites, median log2FC = −1.82) and polyunsaturated acylcarnitines (four metabolites, median log2FC −1.56) (Figure 1E). Conversely, pregnenolone steroids (five metabolites, median log2FC = 1.95) and corticosteroids (four metabolites, median log2FC = 2.04) were significantly increased among VA-ECMO subjects.

Similarly, when VA-ECMO subjects were compared against healthy controls, changes in lipid metabolites predominated, with 137 of 283 significantly altered metabolites (48.4%) being lipids (median log2FC = −1.02) (Supplementary Figure S1B). Categories of lipid metabolites that were decreased among VA-ECMO subjects included SMs (18 metabolites, median log2FC = −1.19), LPLs (12 metabolites, median log2FC = −1.97), and plasmalogens (six metabolites, median log2FC = −1.05). Conversely, MAGs (13 metabolites, log2FC = 2.34) and pregnenolone steroids (eight metabolites, median log2FC = 2.02) were significantly increased among VA-ECMO subjects (Supplementary Table S2).

#### 3.2.2. VV-ECMO vs. Controls

**Figure 2:**
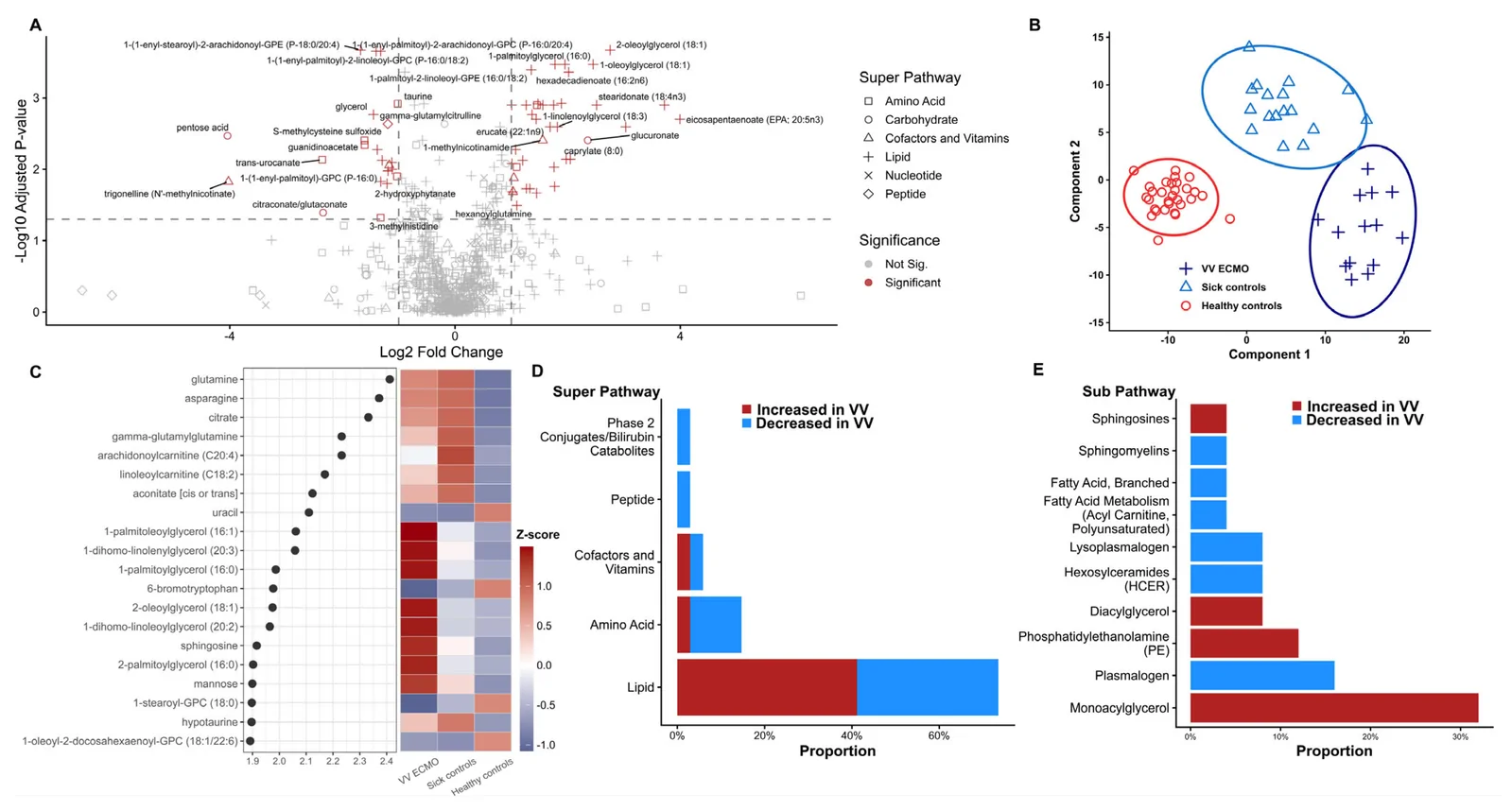
Differential metabolomic signatures in VV-ECMO subjects compared to ICU and healthy controls. (**A**) Volcano plot showing differential metabolite expression between VV-ECMO and sick control groups. Each point represents an individual metabolite, colored by statistical significance (FDR-adjusted *p* < 0.05, |log2 fold change| > 1) with shapes indicating pathway. Lipid metabolites represent the bulk of significant changes. (**B**) Partial Least Squares Discriminant Analysis (PLS-DA) illustrating a clear separation between VV-ECMO, sick controls, and healthy controls. (**C**) Variable importance plot showing that glutamine and asparagine levels are most important in distinguishing the three groups. (**D**) Classes of significantly altered metabolites. Lipid metabolites comprise the majority of both increased and decreased metabolites. (**E**) Categories of significantly altered lipid metabolites demonstrating increases in monoacylglycerols and a decrease in plasmalogens among VV-ECMO subjects.

Comparing VV-ECMO subjects vs. sick controls, 37 of 842 (4.4%) metabolites were significantly different (FC > 2 or <0.5 and q < 0.05) (Supplementary Table S3). Significantly decreased metabolites among VV-ECMO subjects included lipids (plasmalogens, acylcarnitines, SMs, and lysoplasmalogens), bilirubin metabolites (pentose acid), and amino acid metabolites (histidine, creatinine, tryptophan, and methionine metabolism). Conversely, increased metabolites were mostly lipids [MAGs and phosphatidylethanolamine (PE)] (Figure 2A).

When compared to healthy controls, 236 (28.0%) metabolites were significantly different (Supplementary Table S4). Significantly decreased metabolites among VV-ECMO subjects included lipids (LPLs, plasmalogens, SM, and PCs), amino acids (tryptophan, creatinine, histidine, and tyrosine metabolism), and nucleotides (pyrimidine and purine metabolism) (Supplementary Figure S2A). Significantly increased metabolites included TCA cycle metabolites (citrate, aconitate), amino acids (glutamate, alanine, lysine, methionine metabolism), lipids (sphingosines, long-chain PUFAs, MAGs), and carbohydrates (N-acetylglucosamine, mannose).

PLS-DA indicated that three components accounted for optimal differentiation among subject groups. The variance explained by the three components was 13.8%, 4.2%, and 11.6%, while achieving a minimum overall error rate of 0.121 and balanced error rate of 0.154 (Figure 2B). Metabolites with the highest VIP scores were glutamine, asparagine, citrate, arachidonoylcarnitine (C20:4), gamma-glutamylglutamine, and linoleoylcarnitine (C18:2) (Figure 2C).

Lipids constituted the majority of metabolites that were altered in both comparisons. Considering VV-ECMO vs. sick controls, 25 of 37 total significantly altered metabolites (67.6%) were lipids, with a median log2FC of −0.52, with an overall net neutral effect due to some subgroups being increased and others decreased (Figure 2D). Subgroups of lipid metabolites that were decreased among VV-ECMO subjects included plasmalogens (four metabolites, median log2FC = −1.18), lysoplasmalogens (two metabolites, median log2FC = −1.21), hexosylceramides (two metabolites, median log2FC = −1.00), polyunsaturated acylcarnitines (one metabolite, log2FC = −1.19), and SMs (one metabolite, log2FC = −0.73). Conversely, significantly increased metabolites among VV-ECMO subjects included MAGs (eight metabolites, median log2FC = 1.86), PEs (three metabolites, median log2FC = 1.35), and DAGs (two metabolites, median log2FC = 1.01) (Figure 2E).

For VV-ECMO vs. healthy controls, lipid metabolites accounted for 117 of 236 total significantly altered metabolites (49.6%) with a median log2FC of −0.27, as increased and decreased metabolites largely offsetting each other (Supplementary Figure S2B). MAGs (13 metabolites, median log2FC = 3.62), PEs (11 metabolites, median log2FC = 1.52), long-chain PUFAs (six metabolites, median log2FC = 1.16), and DAGs (six metabolites, median log2FC = 0.99) were significantly increased among the VV-ECMO group. Conversely, SMs (10 metabolites, median log2FC = −0.79), LPLs (six metabolites, median log2FC = −0.99), and plasmalogens (seven metabolites, median log2FC = −1.33) were significantly decreased (Supplementary Table S4).

### 3.3. Metabolites Associated with ABI

#### 3.3.1. All ECMO Subjects

In order to identify metabolic shifts occurring prior to the diagnosis of ABI, metabolites at T1 were compared among ECMO subjects (both VA and VV) who developed ABI (n = 7) and those who did not (n = 16). Only three (<1%) metabolites met an FDR-corrected *p*-value for significance (q < 0.05) when comparing all ECMO subjects, including both VA- and VV-ECMO (Supplementary Table S5). They were 1-stearoyl-2-linoleoyl-GPI (18:0/18:2) (q = 0.04, log2FC = −1.45), 1-palmitoyl-2-linoleoyl-GPC (16:0/18:2) (q = 0.04, log2FC = −0.47), and 1-palmitoyl-2-linoleoyl-GPE (16:0/18:2) (q = 0.04, log2FC = −1.38): glycerophospholipids [PC, PE, and phosphatidylinositol (PI)] with a saturated fatty acid at sn-1 and linoleic acid at sn-2. All these glycerophospholipids were decreased in ECMO subjects who developed ABI.

**Figure 3:**
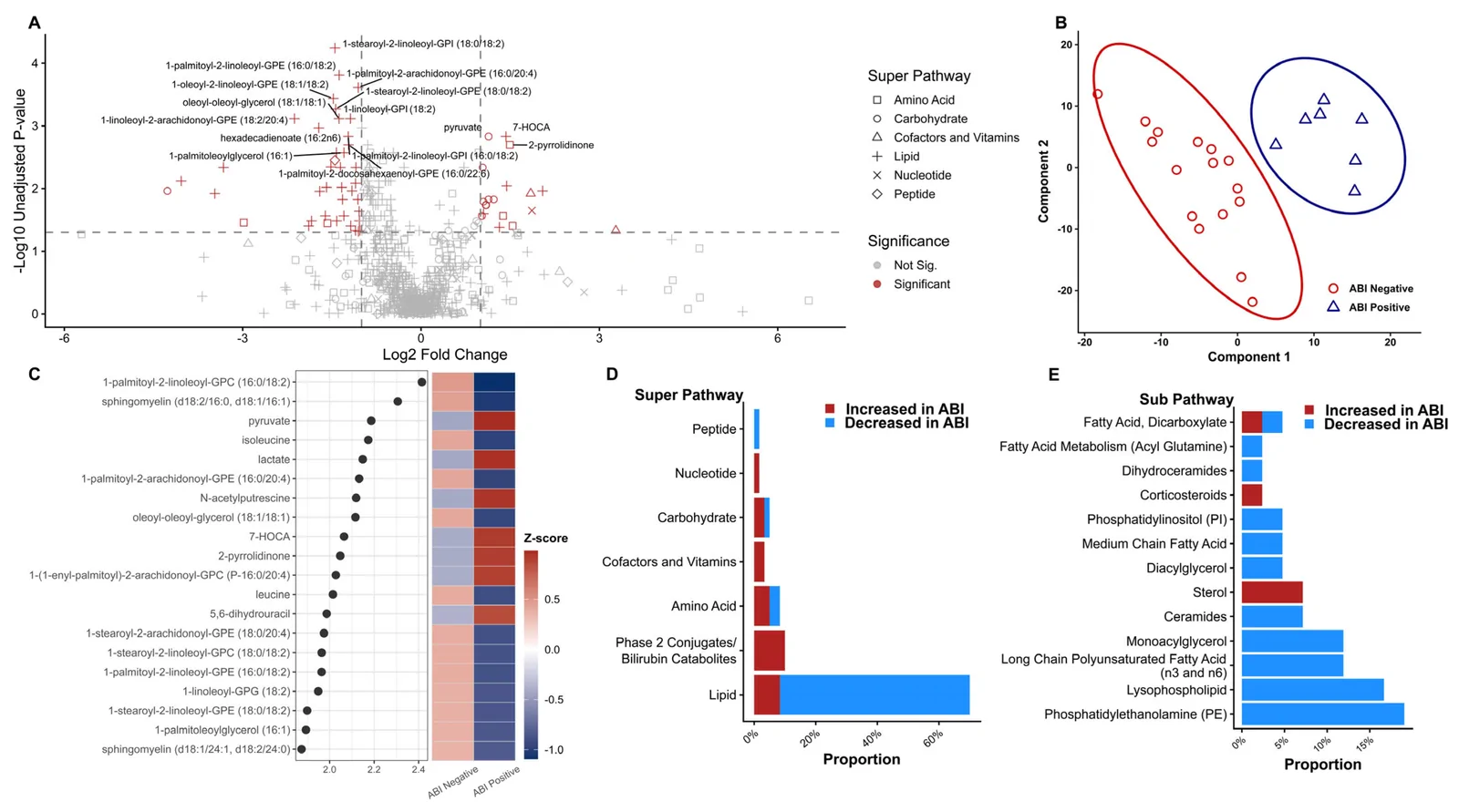
Differential metabolomic signatures in ECMO subjects with and without ABI. (**A**) Volcano plot showing differential metabolite expression between ECMO subjects with (ABI (+)) and without (ABI (−)) acute brain injury (ABI). Each point represents an individual metabolite, colored by statistical significance (unadjusted *p* < 0.05, |log2 fold change| > 1) and shaped by Super Pathway classification. Unadjusted *p*-values were used. Decreases in lipid metabolites are characteristic. (**B**) Partial Least Squares Discriminant Analysis (PLS-DA) illustrating separation between ABI (+) and ABI (−) ECMO subjects. (**C**) Variable importance plot showing increased lactate, 7-HOCA, and amino acid metabolites, and decreased lysophospholipid and phosphatidylethanolamine species are the most distinguishable features in subjects with ABI. (**D**) Distribution of significantly altered metabolites. Lipid metabolites comprise the majority of changes, with most showing a decrease in ABI (+) subjects. (**E**) The distribution of significantly altered lipid metabolites shows a decrease in phosphatidylethanolamines, monoacylglycerols, and long-chain polyunsaturated fatty acids.

Given that only three metabolites met the FDR threshold for significance, metabolites were also ranked according to raw *p*-values. A total of 64 metabolites differed between ECMO subjects with and without ABI (raw *p* < 0.05 and FC > 2 or <0.5) (Figure 3A, Supplementary Table S5). PLS-DA showed modest separation (Figure 3B). It revealed that two components were sufficient to yield minimal classification errors, and minimal cross-validated errors were 0.211 for overall error rates and 0.247 for balanced error rates, with each component explaining approximately 13% of the variance. The five most important metabolites in the classification ranked by their VIP scores were 1-palmitoyl-2-linoleoyl-GPC (16:0/18:2), sphingomyelin (d18:2/16:0, d18:1/16:1), pyruvate, isoleucine, and lactate (Figure 3C).

Of all altered metabolites, 42 (65.6%) were lipids, most of which were decreased among subjects who went on to develop ABI (Figure 3D). Significantly decreased categories of lipids among subjects with ABI included PEs (eight metabolites, log2FC = −1.06), LPLs (seven metabolites, log2FC= −1.00), MAGs (five metabolites, log2FC = −1.10), PUFAs (five metabolites, log2FC = −1.72), ceramides (three metabolites, log2FC = −1.06), and PIs (two metabolites, log2FC = −1.22). Conversely, sterols were significantly increased among subjects with ABI (three metabolites, log2FC = 1.05) (Figure 3E).

#### 3.3.2. VA-ECMO

**Figure 4:**
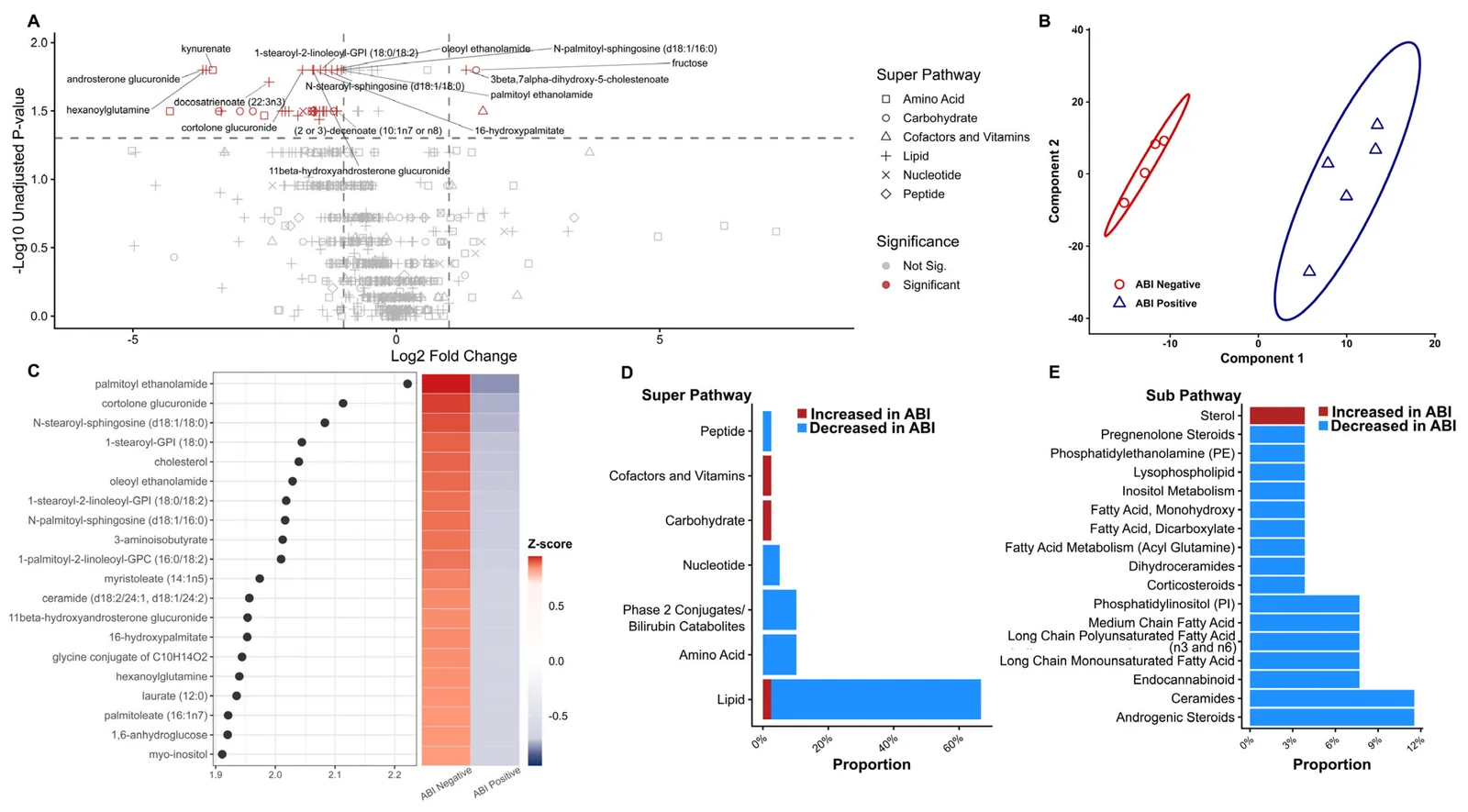
Differential metabolomic signatures in VA-ECMO subjects with and without ABI. (**A**) Volcano plot showing differential metabolite expression between VA-ECMO subjects with (ABI (+)) and without (ABI (−)) acute brain injury (ABI). Each point represents an individual metabolite, colored by statistical significance (unadjusted *p* < 0.05, |log2 fold change| > 1) and shaped by Super Pathway classification. Unadjusted *p*-values were used. (**B**) Partial Least Squares Discriminant Analysis (PLS-DA) illustrating a clear separation between ABI (+) and ABI (−) VA-ECMO subjects. (**C**) Variable importance plot: decrease in lipid species involved in endocannabinoid signaling, steroid, and sphingolipid metabolism is the most distinguishable feature in VA-ECMO subjects with ABI. (**D**) Distribution of significantly altered metabolites. Lipid metabolites comprise the majority of changes, with most showing a decrease in ABI (+) VA-ECMO subjects. (**E**) The distribution of altered lipid metabolites shows a decrease in all subclasses of lipid metabolites except sterols.

Since the majority of subjects who developed ABI were on VA-ECMO support, metabolites associated with ABI were assessed considering only VA-ECMO subjects. No metabolites met the FDR-corrected *p*-value threshold. Considering unadjusted *p*-values, 44 metabolites were altered (raw *p* < 0.05 and FC > 2 or <0.5) (Figure 4A, Supplementary Table S6). PLS-DA revealed that a two-component model showed the best performance throughout repeated cross-validation, with overall error rates of 0.153 and balanced error rates of 0.161 (Figure 4B). The most important variables for classification, evaluated with VIP scores, were palmitoyl ethanolamide, cortolone glucuronide, N-stearoyl-sphingosine (d18:1/18:0), 1-stearoyl-GPI (18:0), cholesterol, and oleoyl ethanolamide (Figure 4C).

Of all altered metabolites, 26 (59.1%) were lipids, with most being decreased in the ABI group (Figure 4D). Numerous classes of lipid metabolites were decreased prior to ABI, including androgenic steroids (three metabolites, median log2FC = −3.32), ceramides (three metabolites, median log2FC = −1.23), endocannabinoids (two metabolites, median log2FC = −1.13), long-chain MUFA (two metabolites, median log2FC = −2.07), long-chain PUFA (two metabolites, median log2FC = −1.97), and medium-chain fatty acids (two metabolites, median log2FC = −1.65) (Figure 4E). Notably, increased levels of one sterol (3beta,7alpha-dihydroxy-5-cholestenoate, median log2FC = 1.32) were observed in subjects who developed ABI.

#### 3.3.3. Shifts in Phospholipids According to ABI Status

**Figure 5:**
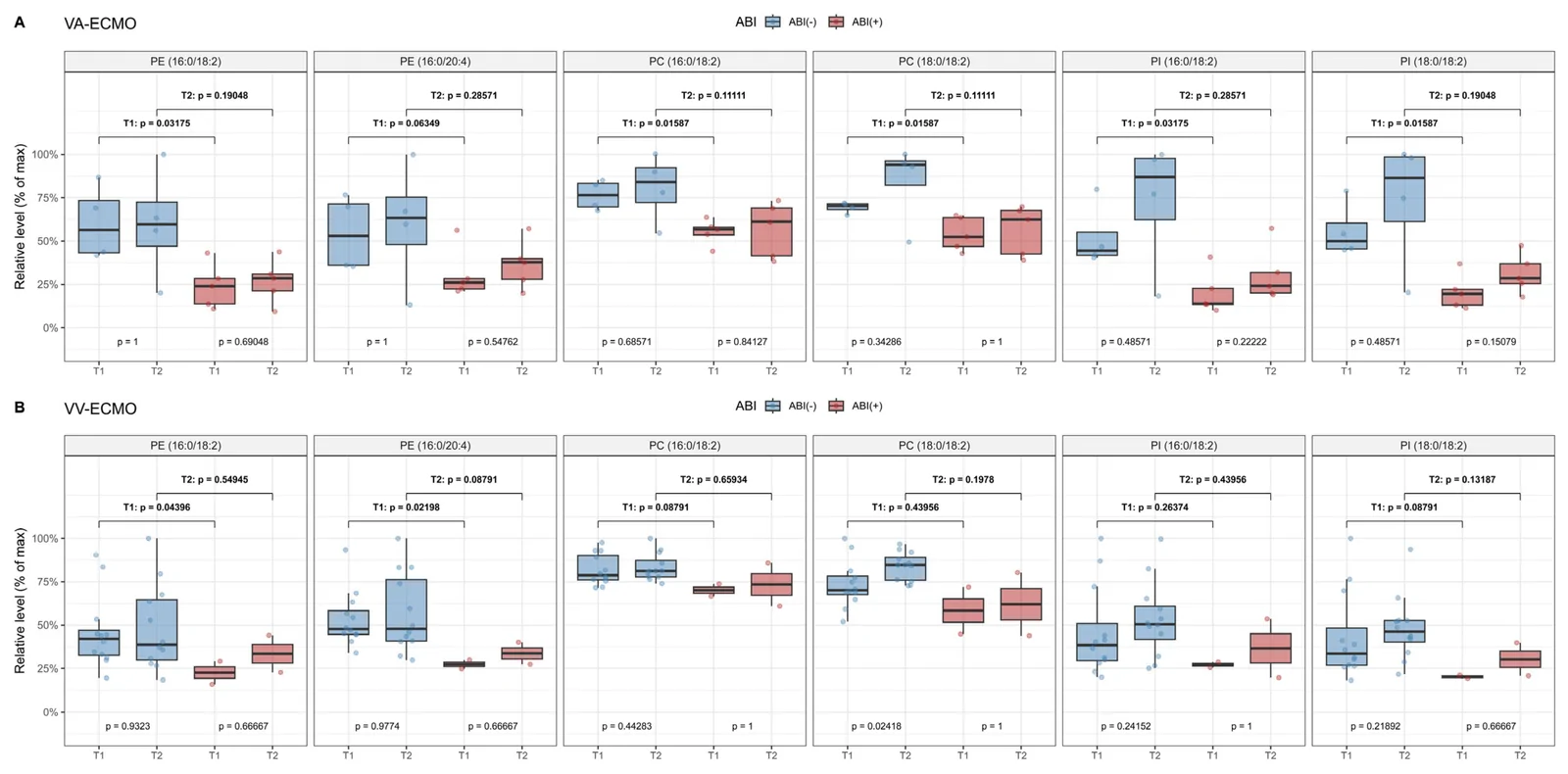
(**A**) Altered phospholipid metabolism in ABI (+) ECMO subjects across timepoints. Boxplots show changes in phosphatidylethanolamine (PE), phosphatidylcholine (PC), and phosphatidylinositol (PI) species at early (T1) and late (T2) timepoints in ABI (−) and ABI (+) VA-ECMO subjects. For each metabolite class, representative metabolites are shown. Temporally dynamic changes in each metabolite are not evident. For all PE species, significant differences are observed at T1 between ABI (−) and ABI (+) in VA-ECMO subjects, as well as for selected PC and PI species. (**B**) In VV-ECMO subjects, a similar trend is observed but not significant. Differences observed at T2 are not significantly associated with ABI in both VA- and VV-ECMO subjects.

Given the significant shifts in phospholipids observed among subjects who developed ABI, temporal trends in different classes of phospholipids were analyzed (Figure 5). Among VA-ECMO subjects at T1, phospholipids including PC (16:0/18:2) (*p* = 0.0159), PC (18:0/18:2) (*p* = 0.0159), PI (18:0/18:2) (*p* = 0.0159), PE (16:0/18:2) (*p* = 0.0317), and PI (16:0/18:2) (*p* = 0.0317) were significantly decreased among those who developed ABI (Figure 5A). On average, subjects who did not develop ABI had 1.28- to 2.71-fold higher expression of key phospholipid metabolites at T1, with the most prominent differences seen for PIs. From T1 to T2, no significant differences were noted among phospholipid metabolites despite some showing a trend toward increasing at T2 (e.g., PIs). Notably, most of the significant differences between subjects with or without ABI at T1 were no longer significant at T2.

Among VV-ECMO subjects, only the PE species remained significantly different at T1 (Figure 5B): PE (16:0/20:4) (*p* = 0.0220) and PE (16:0/18:2) (*p* = 0.0440) were both lower among subjects who developed ABI, with phospholipid levels averaging 1.17- to 2.14-fold lower than those in subjects without ABI. The PC and PI species showed the same directionality but did not reach statistical significance in this small VV-ECMO subset (n = 2 with ABI vs. n = 12 without ABI). No significant changes were noted considering temporal trends from T1 to T2.

## 4. Discussion

In this two-center untargeted metabolomics study, we demonstrate that ECMO support is associated with widespread remodeling of circulating lipid metabolism and that early depletion of structurally related glycerophospholipids precedes the clinical diagnosis of acute brain injury. VA- and VV-ECMO shared a broad ECMO-associated lipidomic signature involving SMs, LPLs, plasmalogens, acylcarnitines, and glycerophospholipids, suggesting that extracorporeal circulation itself contributes to systemic metabolic dysregulation. However, VA-ECMO demonstrated a substantially larger metabolic footprint than VV-ECMO, with more extensive depletion of sphingomyelins, LPLs, and plasmalogens, likely reflecting the greater hemodynamic, inflammatory, and oxidative stress imposed by circulatory support. Across both modes, subjects who developed ABI exhibited early depletion of PEs, PCs, PIs, and LPLs—lipid species central to membrane integrity, mitochondrial function, endothelial stability, and neuroinflammatory signaling within the neurovascular unit. These findings suggest that ECMO-associated ABI is accompanied by an early systemic lipid-remodeling phenotype that may reflect evolving neurovascular vulnerability before overt neurological deterioration.

### 4.1. Mode-Specific Metabolic Signatures

VA- and VV-ECMO were both associated with widespread systemic metabolic remodeling, particularly within lipid pathways. Across ECMO modes, we observed recurrent alterations in SMs, LPLs, plasmalogens, acylcarnitines, and glycerophospholipids, suggesting that extracorporeal circulation itself may contribute to broad lipidomic dysregulation. These shared changes are biologically plausible given the exposure of blood to non-endothelialized circuit surfaces, nonphysiologic shear stress, inflammatory activation, oxidative stress, and altered substrate utilization during critical illness [28,29,30]. Thus, rather than representing entirely distinct metabolic states, VA- and VV-ECMO appear to share a common ECMO-associated lipidomic signature.

Within this shared signature, however, VA-ECMO demonstrated a substantially larger metabolic footprint than VV-ECMO. VA-ECMO subjects showed broader reductions in circulating SMs, LPLs, plasmalogens, and acylcarnitines, whereas VV-ECMO subjects demonstrated a more limited pattern of lipid remodeling, including elevations in monoacylglycerols and select glycerophospholipids. These quantitative differences likely reflect the greater hemodynamic and inflammatory burden imposed by VA-ECMO. Unlike VV-ECMO, which primarily supports gas exchange while preserving native cardiac output, VA-ECMO directly alters systemic and cerebral hemodynamics through nonpulsatile retrograde aortic flow, altered cerebral autoregulation, high shear stress, and nonphysiologic flow patterns [31]. These features may amplify oxidative membrane injury, mitochondrial dysfunction, and systemic inflammatory activation.

The more pronounced lipid depletion observed in VA-ECMO may therefore reflect increased membrane turnover, oxidative stress, and impaired cellular resilience in the setting of cardiogenic shock and systemic hypoperfusion. Plasmalogens serve as endogenous antioxidants through their vinyl ether bond, which preferentially scavenges reactive oxygen species, and their depletion has been described in sepsis and critical illness as a marker of oxidative stress [32,33]. SM hydrolysis by sphingomyelinases is also a well-characterized response to oxidative stress and generates ceramide, a pro-apoptotic and pro-inflammatory mediator [34,35,36]. The concurrent depletion of plasmalogens, SMs, LPLs, and acylcarnitines in VA-ECMO is therefore consistent with a more severe ECMO-associated metabolic stress response rather than a completely distinct metabolic phenotype.

Conversely, the accumulation of monoacylglycerols in VV-ECMO may reflect enhanced lipolysis, altered energy utilization, or the metabolic consequences of prolonged respiratory failure. Most VV-ECMO subjects in this cohort required ECMO for COVID-19–associated ARDS, and SARS-CoV-2 infection itself has been associated with plasmalogen depletion and lipid metabolic alterations [32]. These findings highlight the importance of considering both ECMO mode and underlying disease state when interpreting circulating metabolomic profiles. Overall, ECMO mode appears to modify the magnitude and pattern of lipidomic remodeling, with VA-ECMO producing a larger and more severe metabolic footprint superimposed on a shared extracorporeal circulation-associated signature.

### 4.2. Systemic Inflammation and Metabolic Disruption During ECMO

The shared metabolic perturbations observed across VA- and VV-ECMO should be interpreted in the context of the systemic inflammatory response triggered by extracorporeal circulation. Exposure of blood to the non-endothelialized surfaces of the ECMO circuit activates the complement system, contact system, and coagulation cascade, resulting in the release of pro-inflammatory cytokines including IL-1β, IL-6, IL-8, and TNF-α [28,29,30]. The oxygenator membrane has been identified as a major driver of leukocyte activation and TNF-α elevation during extracorporeal circulation [28]. This inflammatory milieu activates neutrophils and promotes neutrophil extracellular trap formation, which has been observed in ECMO patients and within circuit-associated thrombi [37,38,39]. NETs may contribute to blood–brain barrier disruption by degrading tight junction proteins and activating inflammatory cascades, thereby linking systemic circuit-driven inflammation to neurovascular vulnerability [40,41].

These established inflammatory pathways provide a biologically plausible context for interpreting the phospholipid depletion observed in our study. Pro-inflammatory cytokines, particularly TNF-α and IL-1β, can stimulate phospholipase A2 activity and sphingomyelinase-mediated ceramide generation [36,42,43]. Hydrolysis of membrane phospholipids releases arachidonic acid and LPLs, which may subsequently participate in pro-inflammatory lipid signaling [42,43]. Accordingly, the broad reductions in circulating phospholipids observed across ECMO subjects, and the more pronounced depletion among those who developed ABI, are consistent with altered phospholipid remodeling in the setting of systemic inflammation and neurovascular stress. However, inflammatory mediators and phospholipase activity were not directly measured in this study, and these pathways should therefore be considered mechanistic hypotheses for the observed metabolomic changes.

### 4.3. Phospholipid Depletion, Neuroinflammation, and ABI

Across ECMO modes, early reductions in a small set of structurally related glycerophospholipids were consistently associated with subsequent ABI. The three metabolites meeting FDR-corrected significance [1-stearoyl-2-linoleoyl-GPI (18:0/18:2), 1-palmitoyl-2-linoleoyl-GPC (16:0/18:2), and 1-palmitoyl-2-linoleoyl-GPE (16:0/18:2)] share a common architecture: a saturated fatty acid at the sn-1 position and linoleic acid at the sn-2 position. This conserved structure is noteworthy because the sn-2 position is precisely the bond cleaved by PLA2 [44,45]. The coordinated depletion of these structurally related species is therefore consistent with altered PLA2-associated phospholipid remodeling and raises the possibility that this pathway contributes to the observed lipidomic signature.

PLA2 activation is a central event in cerebral ischemia-reperfusion injury. Cytosolic PLA2 (cPLA2) is rapidly phosphorylated and activated via the p38 MAPK pathway during ischemia, directly contributing to blood–brain barrier disruption, cerebral edema, and oxidative injury [46]. In the ischemic penumbra, secretory PLA2 (sPLA2-X) expressed by neurons and astrocytes generates lysophosphatidylcholine (LPC), which activates microglia via G2A and P2X7 receptors, upregulating MCP-1/CCR2-mediated chemotaxis and amplifying neuroinflammation [47,48]. Products of PLA2-mediated phospholipid hydrolysis, including free fatty acids and LPLs, may also serve as substrates or intermediates in the generation of bioactive inflammatory lipid mediators [42,43,44].

Linoleic acid released during phospholipid remodeling can undergo oxidation to hydroxyoctadecadienoic acids (HODEs), which have been associated with pro-inflammatory signaling, including activation of NF-κB–related pathways in vascular endothelial cells [49,50]. In this context, the preferential depletion of linoleic acid-containing phospholipids observed in subjects who subsequently developed ABI is compatible with increased phospholipid remodeling and altered inflammatory lipid signaling.

LPLs, the other products of PLA2-catalyzed hydrolysis, are themselves potent neuroinflammatory mediators. LPC activates caspase-1 through a novel pathway involving both NLRP3 and NLRC4 inflammasomes in microglia, triggering IL-1β release and amplifying neuroinflammatory cascades [51,52]. LPC also induces microglial chemotaxis, astrocyte activation, and neuronal loss through Rho kinase-dependent mechanisms [48]. In ischemic brain tissue, lipidomic studies have demonstrated an imbalanced ratio between PC and LPC, driven by the Lands cycle, with LPC directly impairing neuronal growth cones and viability while intact PC suppresses microglial secretion of IL-1β and TNF-α [53]. In our cohort, circulating LPLs were decreased among subjects who developed ABI. Although this finding could be consistent with altered phospholipid turnover or redistribution during evolving tissue injury, this study cannot determine whether the lower circulating concentrations reflect local consumption, altered synthesis, increased clearance, or other changes in lipid metabolism. Notably, decreased circulating LPLs have also been associated with worse outcomes after traumatic brain injury and subarachnoid hemorrhage, supporting the biological plausibility of an association between reduced circulating LPLs and neurological injury [23,24].

### 4.4. SM–Ceramide Axis and Neuroinflammatory Amplification

The reductions in circulating sphingomyelins observed in subjects who developed ABI are consistent with alterations in sphingolipid metabolism that may be relevant to neuroinflammatory signaling. SM hydrolysis by neutral and acid sphingomyelinases generates ceramide, a bioactive sphingolipid that serves as a critical second messenger in inflammation, apoptosis, and cell death [35,54,55]. In cerebral ischemia, ceramide levels are markedly elevated through both de novo synthesis and SM hydrolysis, and ceramide accumulation has been directly linked to exacerbation of ischemic injury [55,56,57].

Ceramide exerts its neuroinflammatory effects through multiple mechanisms. In astrocytes, ceramide increases mitochondrial permeabilization, triggering the release of mitochondrial DNA into the cytoplasm and activating the cGAS/STING pathway, which induces interferon responses that aggravate inflammatory damage in the ischemic brain [56]. Early activation of the neutral sphingomyelinase 2 (nSMase2)/ceramide pathway in astrocytes has been shown to upregulate TNF-α, IL-1β, and IL-6, contributing to neuronal damage via inflammatory mechanisms [58]. Ceramide also accumulates in mitochondria, where it causes respiratory chain dysfunction through JNK3-dependent activation of ceramide synthase—a process that is abolished in JNK3-deficient mice, which exhibit reduced infarct volumes after ischemia-reperfusion [57].

Importantly, cytokine-mediated SM degradation to ceramide is redox-sensitive: TNF-α and IL-1β deplete intracellular glutathione and promote sphingomyelin hydrolysis, creating a feed-forward loop in which oxidative stress drives ceramide generation, which in turn amplifies neuroinflammation [36]. In our cohort, decreased circulating SMs among subjects who developed ABI are consistent with altered sphingolipid metabolism in the setting of neurological injury. However, because ceramide concentrations and sphingomyelinase activity were not directly measured, the present data cannot determine whether lower circulating SMs reflect increased conversion to ceramide, altered synthesis, redistribution, or other changes in sphingolipid turnover.

### 4.5. Plasmalogen Depletion and Loss of Anti-Inflammatory Protection

Plasmalogens were significantly decreased in both VA- and VV-ECMO subjects, with more pronounced reductions in those who developed ABI. Beyond their role as antioxidant scavengers, plasmalogens actively inhibit neuroinflammation and promote cognitive function [59]. Inflammatory stimuli reduce plasmalogen levels in glial cells via NF-κB activation, which downregulates the plasmalogen-synthesizing enzyme glycerone phosphate O-acyltransferase (GNPAT) through increased c-Myc recruitment [60]. Critically, plasmalogen reduction itself activates NF-κB in glial cells and microglia, creating a vicious cycle in which inflammation depletes plasmalogens, and plasmalogen depletion further accelerates neuroinflammation [59,60]. This bidirectional relationship has been demonstrated in lipopolysaccharide-treated mice, aged brains, and postmortem human Alzheimer’s disease brain tissue [60]. In the context of ECMO, where systemic inflammation is driven by circuit–blood interactions, plasmalogen depletion may represent both a consequence of and a contributor to neuroinflammatory injury.

Plasmalogen hydrolysis also releases arachidonic acid, which serves as a precursor for pro-inflammatory prostaglandins, leukotrienes, and thromboxanes [61,62]. The concurrent depletion of plasmalogens and PUFAs observed in our ABI subjects may therefore reflect both increased substrate consumption for pro-inflammatory mediator synthesis and loss of the anti-inflammatory and pro-resolving capacity normally provided by these lipid species.

### 4.6. PUFA Depletion and Impaired Resolution of Neuroinflammation

Polyunsaturated fatty acids, particularly docosahexaenoic acid (DHA) and eicosapentaenoic acid (EPA), are critical for the resolution of neuroinflammation. DHA is metabolized to specialized pro-resolving mediators (SPMs), including resolvins, protectins, and maresins, which actively suppress microglial pro-inflammatory responses, inhibit NF-κB signaling, and promote the transition from pro-inflammatory to anti-inflammatory microglial phenotypes [63,64,65]. Unesterified DHA has been shown to attenuate neuroinflammation in lipopolysaccharide-initiated models, at least in part through conversion to neuroprotectin D1 [65]. The depletion of PUFAs observed in ECMO subjects who developed ABI (median log2FC = −1.72) is consistent with an altered balance of lipid mediators involved in inflammatory regulation and resolution.

Additionally, omega-3 PUFAs maintain blood–brain barrier integrity and support glymphatic function [66]. In this context, the lower circulating PUFA levels observed during ECMO may reflect a reduction in lipid species associated with neurovascular and anti-inflammatory homeostasis. However, this study did not directly assess blood–brain barrier integrity or glymphatic function, and therefore future studies will need to determine whether PUFA depletion contributes to impairment of these protective mechanisms.

### 4.7. Endocannabinoid Depletion and Neuroinflammatory Vulnerability

Among VA-ECMO subjects who developed ABI, endocannabinoids (palmitoyl ethanolamide and oleoyl ethanolamide) were among the most important metabolites for classification by VIP scores, and both were significantly decreased. The endocannabinoid system plays a critical neuroprotective role after brain injury. Endocannabinoids (2-arachidonoylglycerol and N-arachidonoylethanolamine) are anti-inflammatory lipid mediators that temper microglial pro-inflammatory responses and promote the transition to anti-inflammatory phenotypes [67,68,69]. In traumatic brain injury models, enhancing endocannabinoid signaling in astrocytes reduces neuropathology and prevents synaptic and cognitive decline, while endocannabinoid depletion exacerbates neurovascular injury and compromises brain–CSF barrier integrity [67,70].

Pharmacological blockade of endocannabinoid receptors during acute brain injury lengthens hippocampal neuroinflammation and reverses the upregulation of anti-inflammatory molecules, supporting an important role for endocannabinoid signaling in initiating adaptive anti-inflammatory processes [71]. In our cohort, the depletion of endocannabinoids observed in VA-ECMO subjects who developed ABI is consistent with altered endogenous lipid signaling in the setting of neurological injury. However, this study cannot determine whether reduced circulating endocannabinoid levels directly impaired neuroprotective signaling or increased susceptibility to ECMO-associated inflammatory stress.

### 4.8. Spectrum of ABI and the Shared Lipidomic Signature

The spectrum of ABI observed in this cohort (ischemic stroke [n = 4], HIBI [n = 1], IPH [n = 1], and SAH [n = 1]) is consistent with the known epidemiology of ECMO-associated neurological injury. A systematic review and meta-analysis of 16,063 ECMO patients found that VA-ECMO carries higher rates of ischemic stroke (10% vs. 1%), HIBI (13% vs. 1%), and brain death (11% vs. 1%) compared with VV-ECMO, while rates of intracerebral hemorrhage were similar between modes (6% vs. 8%) [4]. The predominance of ischemic events in the VA-ECMO subgroup of this study is consistent with these data and may be related to the distinct hemodynamic disturbances associated with VA-ECMO, including reduced pulsatility, retrograde aortic flow, and altered cerebral perfusion [1,13,31]. Importantly, neurological risk may not be proportional to the cumulative duration of ECMO support. Although VV-ECMO subjects in our cohort had substantially longer ECMO runs, this likely reflects the prolonged course of severe respiratory failure, whereas VA-ECMO is commonly initiated in the setting of severe circulatory instability and may expose the brain to greater hemodynamic and embolic risk early after cannulation. ECMO duration may also be influenced by competing risks, as patients who develop severe early ABI may die or undergo withdrawal of support before accumulating a prolonged ECMO course.

Despite the heterogeneity of ABI subtypes, a shared lipidomic signature was observed across injury types. This pattern is consistent with metabolomic findings in other forms of acute brain injury. Ischemic stroke studies have reported reductions in circulating PCs and SMs alongside broader alterations in phospholipid and sphingolipid metabolism [72,73,74]. In TBI, choline-containing phospholipids, including LPCs, ether PCs, and SMs, have been associated with injury severity and functional outcome [24,75]. Notably, higher circulating linoleic acid-containing lysophospholipids have been associated with better outcomes after mild TBI [22], consistent with the broader association between reduced 18:2-containing phospholipids and subsequent ABI observed in this study. Similarly, decreased circulating lysophospholipids have been associated with worse functional outcomes after aneurysmal subarachnoid hemorrhage [23]. Together, these observations support external consistency of the phospholipid alterations identified during ECMO and suggest that altered membrane lipid homeostasis may represent a shared feature of cerebral vulnerability across different forms of acute brain injury. Notably, these lipid metabolites differ from many established and emerging neurotrauma biomarkers, which predominantly include proteins reflecting neuronal, astroglial, or axonal injury [76]. Metabolomic signatures may therefore provide complementary information regarding membrane remodeling, systemic metabolic stress, and inflammatory processes that are not directly captured by conventional protein biomarkers. However, this study did not directly compare metabolomic and protein biomarkers, and future studies will be required to determine whether these approaches provide independent or additive prognostic information.

### 4.9. Temporal Dynamics and the Window of Vulnerability

The phospholipid differences between subjects with and without ABI were most pronounced within 24 h of cannulation and were no longer significant at later timepoints. This temporal pattern raises the possibility that early metabolic shifts may identify a period of neurological vulnerability before ABI becomes clinically or radiographically recognized. Such a period is particularly relevant during ECMO, when sedation and neuromuscular blockade may limit neurological examination, CT has limited sensitivity for early ischemic injury, and conventional MRI is often impractical during ECMO support. Although emerging modalities such as ultra-low-field portable MRI have demonstrated feasibility for ABI detection during ECMO, they provide intermittent rather than continuous assessment [77]. Serial blood-based metabolomic profiling could therefore complement neurological examination and neuroimaging by providing additional information for early neurological risk stratification.

### 4.10. Clinical Implications

These findings have several potential implications. First, plasma metabolomic profiling may provide an objective, noninvasive means of identifying ECMO patients at heightened neurological risk when traditional assessments are limited. The identification of a small set of structurally related phospholipids that precede ABI diagnosis could form the basis for a targeted biomarker panel amenable to rapid clinical assays. Second, the observed phospholipid alterations are consistent with perturbations in biological pathways involving PLA2-mediated phospholipid remodeling, ceramide metabolism, plasmalogens, and endocannabinoid signaling. Although these mechanisms were not directly evaluated in this study, they provide testable hypotheses and identify candidate pathways for future mechanistic investigation. Strategies targeting membrane integrity, phospholipid metabolism, or inflammatory signaling may therefore warrant evaluation in experimental models before consideration as neuroprotective interventions during ECMO support [78]. Third, integrating metabolomic data with physiologic monitoring, anticoagulation parameters, and neuroimaging could ultimately enable more precise neurological risk stratification and individualized management strategies.

## 5. Limitations

This study has several limitations. First, the number of subjects who developed ABI was modest (n = 7), limiting statistical power. Most ABI-associated metabolites did not meet predefined FDR thresholds, likely reflecting this small sample size. To mitigate this limitation, complementary approaches including fold-change analysis, VIP rankings, pathway-level aggregation, and temporal trends were employed, which revealed consistent biological signals despite limited power. Second, VA- and VV-ECMO subjects differed significantly in age (67.4 vs. 38.7 years), underlying diagnoses (cardiogenic shock vs. COVID-19 ARDS), and ECMO duration (11.3 vs. 35.9 days), making it difficult to attribute metabolic differences solely to ECMO mode versus the underlying disease state. The VV-ECMO cohort being predominantly COVID-19 patients introduces additional confounding, as SARS-CoV-2 infection itself causes lipid metabolic alterations [32]. Third, the heterogeneity of ABI subtypes precluded subtype-specific analyses. Fourth, untargeted metabolomics provides relative rather than absolute quantification and cannot directly establish causality. Fifth, although patients were effectively fasting/NPO at the initial sampling time point used for the primary analyses, nutritional exposure was heterogeneous at T2 and may have contributed to variability in longitudinal metabolomic measurements. Finally, external validation in larger, multicenter cohorts will be required before clinical implementation.

## 6. Conclusions

ECMO support is associated with substantial, mode-specific metabolic remodeling. Across VA- and VV-ECMO, early depletion of key membrane phospholipids was associated with subsequent acute brain injury, suggesting that disrupted lipid homeostasis may characterize a period of neurological vulnerability before overt injury is recognized. The lipidomic signature associated with ABI is consistent with alterations in pathways involving phospholipid remodeling, ceramide metabolism, plasmalogen depletion, and endocannabinoid signaling. Although these findings cannot establish causal mechanisms, they generate testable hypotheses regarding biological processes that may accompany neurological vulnerability during ECMO support. These results support further investigation of metabolomic profiling for early neurological risk stratification and of the identified pathways in dedicated mechanistic studies.

## Figures and Tables

**Table 1:** Patient Demographics.

Variable		Healthy Control	Sick Control	All ECMO	VA-ECMO	VV-ECMO	*p*-Value ^†^
		n = 30	n = 17	n = 23	n = 9	n = 14	
Age *		59.5 (8.8)	57.9 (17.0)	50.0 (17.5)	67.4 (11.9)	38.7 (9.1)	8.08 × 10^−6^
Sex ^ψ^	Male	15 (50.0%)	10 (58.8%)	15 (65.2%)	5 (55.6%)	10 (71.4%)	0.636
	Female	15 (50.0%)	7 (41.2%)	8 (34.8%)	4 (44.4%)	4 (28.6%)	
Race ^ψ^	Black	8 (26.7%)	6 (35.3%)	6 (26.1%)	3 (33.3%)	3 (21.4%)	7.19 × 10^−5^
	White	21 (70.0%)	5 (29.4%)	16 (69.6%)	6 (66.7%)	10 (71.4%)	
	Asian	1 (3.3%)	0 (0.0%)	1 (4.3%)	0 (0.0%)	1 (7.1%)	
	Other	0 (0.0%)	6 (35.3%)	0 (0.0%)	0 (0.0%)	0 (0.0%)	
Ethnicity ^ψ^	Hispanic	6 (20.0%)	4 (23.5%)	8 (34.8%)	0 (0.0%)	8 (57.1%)	0.007
Reason for ICU Admission ^ψ^	COPD/Asthma Flare		8 (47.1%)				
	PNA		6 (35.3%)				
	Sepsis		4 (23.5%)				
	CHF		4 (23.5%)				
	AKI		2 (11.8%)				

* For numeric variables, mean (standard deviation) is shown in parentheses. ^†^ *p*-values were computed comparing healthy, sick controls, VA-ECMO, and VV-ECMO subjects. ^ψ^ For categorical variables, percentage (%) is shown in parentheses. Abbreviations: COPD (chronic obstructive pulmonary disease), PNA (pneumonia), CHF (congestive heart failure), AKI (acute kidney injury), ICU (intensive care unit).

**Table 2:** Baseline Clinical Characteristics and Cannulation Parameters of ECMO Subjects.

Variable		VA-ECMO	VV-ECMO	*p*-Value
		n = 9	n = 14	
Days on ECMO *		11.3 (11.7)	35.9 (30.0)	0.002
Cannulation	Central	3	0	0.047
	Peripheral	6	14	
ECMO Indication	ARDS	1 (11.1%)	13 (92.9%)	<0.001
	Cardiogenic shock	5 (55.6%)	1 (7.1%)	
	Post-cardiotomy shock	3 (33.3%)	0 (0.0%)	
Pump Flow Rate (L/min) *		3.8 (0.5)	4.2 (0.6)	0.072
Before cannulation *	pH	7.4 (0.1)	7.2 (0.1)	0.021
	Bicarbonate (mmol/L)	20.2 (4.4)	30.5 (10.1)	0.009
	PaO_2_ (mmHg)	179.8 (86.5)	66.4 (22.2)	<0.001
	PaCO_2_ (mmHg)	32.9 (8.3)	76.9 (31.0)	<0.001
	Lactate (mmol/L)	6.9 (6.6)	4.0 (5.0)	0.432
	SaO_2_ (%)	98.0 (2.8)	83.1 (10.7)	<0.001
After cannulation *	pH	7.5 (0.1)	7.4 (0.0)	0.082
	Bicarbonate (mmol/L)	21.2 (3.8)	35.0 (6.4)	<0.001
	PaO_2_ (mmHg)	188.3 (75.7)	69.9 (20.9)	<0.001
	PaCO_2_ (mmHg)	30.9 (9.1)	58.6 (9.4)	<0.001
	Lactate (mmol/L)	7.3 (6.5)	1.4 (0.6)	0.021
	SaO_2_ (%)	99.0 (0.8)	89.5 (9.3)	<0.001
Acute Brain Injury		5 (55.6%)	2 (14.3%)	0.066
Type of ABI	Ischemic stroke	4		0.143
	HIBI	1		
	IPH		1	
	SAH		1	
Time from cannulation to ABI diagnosis		110.9 (48–146.8)	789.2 (720.0–858.4)	0.381
Mortality		8 (88.9%)	8 (57.1%)	0.176

* For numeric variables, mean (standard deviation) is shown. Time from cannulation to ABI diagnosis is displayed as median (IQR). Abbreviations: ARDS (Acute Respiratory Distress Syndrome), HIBI (hypoxic-ischemic brain injury), IPH (intraparenchymal hemorrhage), SAH (subarachnoid hemorrhage).

## Data Availability

Data will be made available upon reasonable request.

## Supplementary Materials

The following supporting information can be downloaded at: https://www.mdpi.com/article/10.3390/cells15171593/s1, Table S1: Significantly altered metabolites in VA-ECMO subjects compared with sick controls; Table S2: Significantly altered metabolites in VA-ECMO subjects compared with healthy controls; Figure S1: VA-ECMO vs. healthy controls; Table S3: Significantly altered metabolites in VV-ECMO subjects compared with sick controls; Table S4: Significantly altered metabolites in VV-ECMO subjects compared with healthy controls; Figure S2: VV-ECMO vs. healthy controls; Table S5: Significantly altered metabolites comparing all subjects with or without ABI; Table S6: Significantly altered metabolites comparing VA-ECMO subjects with or without ABI.
