# Supplementary material for: Early Metabolomic Changes During Extracorporeal Membrane Oxygenation Are Associated with Subsequent Acute Brain Injury
Source: Cells. 2026 Sep 1;15(17):1593. doi: 10.3390/cells15171593 (PMC13565166; doi:10.3390/cells15171593)
Supplement: Supplementary file 1 [file cells-15-01593-s001.zip › cells-4446002-supplementary.pdf]

Supplementary Material

I. **Supplementary Table S1:** Significantly altered metabolites in VA-ECMO subjects compared with sick controls

II. **Supplementary Table S2:** Significantly altered metabolites in VA-ECMO subjects compared with healthy controls

III. **Supplementary Figure S1:** VA-ECMO vs healthy controls

IV. **Supplementary Table S3:** Significantly altered metabolites in VV-ECMO subjects compared with sick controls

V. **Supplementary Table S4:** Significantly altered metabolites in VV-ECMO subjects compared with healthy controls

VI. **Supplementary Figure S2:** VV-ECMO vs healthy controls

VII. **Supplementary Table S5:** Significantly altered metabolites comparing all subjects with or without ABI

VIII. **Supplementary Table S6:** Significantly altered metabolites comparing VA-ECMO subjects with or without ABI

Metabolite	log2fc	p.value	p.adj	SUPER_PATHWAY
biliverdin	4.09563405	6.40E-07	0.00026948	Cofactors and Vitamins
citrate	-0.3065273	6.40E-07	0.00026948	Energy
mannitol/sorbitol	4.93801337	1.28E-06	0.00026948	Carbohydrate
2-pyrrolidinone	2.10260113	2.56E-06	0.00035931	Amino Acid
sphingomyelin (d18:2/24:2)*	-1.2243507	2.56E-06	0.00035931	Lipid
sphingomyelin (d18:1/18:1, d18:2/18:0)	-0.958007	4.48E-06	0.00053896	Lipid
21-hydroxypregnenolone monosulfate (2)	2.83894793	5.23E-06	0.00055038	Lipid
sphingomyelin (d18:1/20:1, d18:2/20:0)*	-1.0688454	7.68E-06	0.00064675	Lipid
thyroxine	-1.4087765	7.68E-06	0.00064675	Amino Acid
1-stearoyl-GPC (18:0)	-2.0736355	1.22E-05	0.00093093	Lipid
sphingomyelin (d18:2/24:1, d18:1/24:2)*	-0.9761306	1.92E-05	0.00124375	Lipid
stearoyl sphingomyelin (d18:1/18:0)	-1.0392886	1.92E-05	0.00124375	Lipid
1-palmitoyl-2-arachidonoyl-GPC (16:0/20:4n6)	-0.480284	2.88E-05	0.00151582	Lipid
1-stearoyl-2-oleoyl-GPS (18:0/18:1)	1.30078285	2.88E-05	0.00151582	Lipid
sphingomyelin (d18:2/23:0, d18:1/23:1, d17:1/24:1)*	-1.0647672	2.88E-05	0.00151582	Lipid
1-palmitoyl-GPC (16:0)	-1.8202463	4.29E-05	0.00171953	Lipid
2-palmitoyl-GPC (16:0)*	-2.1413448	4.29E-05	0.00171953	Lipid
palmitoyl sphingomyelin (d18:1/16:0)	-0.5635329	4.29E-05	0.00171953	Lipid
sphingomyelin (d18:1/24:1, d18:2/24:0)*	-1.0511272	4.29E-05	0.00171953	Lipid
gamma-glutamylcitrulline*	-1.4892674	6.21E-05	0.00217829	Peptide
sphingosine 1-phosphate	-1.3989063	6.21E-05	0.00217829	Lipid
1-(1-enyl-palmitoyl)-GPC (P-16:0)*	-2.5479836	7.11E-05	0.00239359	Lipid
bilirubin degradation product, C17H20N2O5 (2)**	2.21432886	8.83E-05	0.00265629	Phase 2 Conjugates/Bilirubin Catabolites
N-acetylputrescine	1.46490253	8.83E-05	0.00265629	Amino Acid
sphingomyelin (d18:1/22:1, d18:2/22:0, d16:1/24:1)*	-0.9317415	8.83E-05	0.00265629	Lipid
1-arachidonoyl-GPC (20:4n6)*	-1.5060147	0.0001229	0.00323375	Lipid
1-palmitoleoyl-GPC (16:1)*	-1.9882398	0.0001229	0.00323375	Lipid
bilirubin degradation product, C17H20N2O5 (1)**	2.25690402	0.0001229	0.00323375	Phase 2 Conjugates/Bilirubin Catabolites
sphingomyelin (d18:2/16:0, d18:1/16:1)*	-0.7911206	0.0001229	0.00323375	Lipid
4-acetamidobutanoate	1.63955111	0.00016962	0.00396733	Amino Acid
lactosyl-N-palmitoyl-sphingosine (d18:1/16:0)	-0.7752841	0.00016962	0.00396733	Lipid
myo-inositol	1.71044692	0.00016962	0.00396733	Lipid
sphingomyelin (d18:2/23:1)*	-1.1340013	0.00016962	0.00396733	Lipid
chenodeoxycholate	-4.2794025	0.000223	0.00471917	Lipid
3-(4-hydroxyphenyl)lactate (HPLA)	1.11419195	0.00022979	0.00471917	Amino Acid
glucuronate	2.38004073	0.00022979	0.00471917	Carbohydrate
sphingomyelin (d18:2/21:0, d16:2/23:0)*	-1.1309908	0.00022979	0.00471917	Lipid
taurine	-0.9314096	0.00022979	0.00471917	Amino Acid
sphinganine-1-phosphate	-1.5365351	0.00030419	0.00564734	Lipid
1-(1-enyl-palmitoyl)-2-arachidonoyl-GPC (P-16:0/20:4)*	-0.9449907	0.00030852	0.00564734	Lipid
sphingomyelin (d18:1/17:0, d17:1/18:0, d19:1/16:0)	-1.0128223	0.00030852	0.00564734	Lipid
sphingomyelin (d18:1/22:2, d18:2/22:1, d16:1/24:2)*	-0.9378551	0.00030852	0.00564734	Lipid

sphingomyelin (d18:1/25:0, d19:0/24:1, d20:1/23:0, d19:1/24:0)*	-1.2813885	0.00030852	0.00564734	Lipid
lignoceroyl sphingomyelin (d18:1/24:0)	-1.3508113	0.00040902	0.00688788	Lipid
pregnenolone sulfate	2.22637446	0.00040902	0.00688788	Lipid
sphingomyelin (d18:1/20:0, d16:1/22:0)*	-0.9959192	0.00040902	0.00688788	Lipid
stearoylcarnitine (C18)	-0.7636625	0.00040902	0.00688788	Lipid
adrenoylcarnitine (C22:4)*	-1.715879	0.00052558	0.00808436	Lipid
1-oleoyl-GPC (18:1)	-1.3456159	0.00053768	0.00808436	Lipid
1,5-anhydroglucitol (1,5-AG)	-1.4193137	0.00053768	0.00808436	Carbohydrate
behenoyl sphingomyelin (d18:1/22:0)*	-1.0775767	0.00053768	0.00808436	Lipid
1-stearoyl-2-arachidonoyl-GPC (18:0/20:4)	-0.4651988	0.00069898	0.00949261	Lipid
adenine	0.81447635	0.00069898	0.00949261	Nucleotide
arachidonoylcarnitine (C20:4)	-1.5578906	0.00069898	0.00949261	Lipid
isovalerate (C5)	-1.357833	0.00069898	0.00949261	Amino Acid
sphingomyelin (d18:0/18:0, d19:0/17:0)*	-1.6829298	0.00069898	0.00949261	Lipid
tricosanoyl sphingomyelin (d18:1/23:0)*	-1.1797179	0.00069898	0.00949261	Lipid
dihomo-linolenoylcarnitine (C20:3n3 or 6)*	-1.6831703	0.00083125	0.01093615	Lipid
palmitoyl-sphingosine-phosphoethanolamine (d18:1/16:0)	-0.6915487	0.00090189	0.0113342	Lipid
sphingomyelin (d18:1/19:0, d19:1/18:0)*	-1.1093423	0.00090189	0.0113342	Lipid
1-(1-enyl-stearoyl)-2-arachidonoyl-GPE (P-18:0/20:4)*	-1.1961485	0.00115217	0.01328937	Lipid
1-stearoyl-2-linoleoyl-GPC (18:0/18:2)*	-0.2844604	0.00115217	0.01328937	Lipid
alpha-ketoglutarate	1.25205561	0.00115217	0.01328937	Energy
bilirubin degradation product, C17H18N2O4 (3)**	2.16454303	0.00115217	0.01328937	Phase 2 Conjugates/Bilirubin Catabolites
dihomo-linoleoylcarnitine (C20:2)*	-1.2749474	0.00115217	0.01328937	Lipid
kynurenate	2.85812828	0.00115217	0.01328937	Amino Acid
bilirubin (E,Z or Z,E)*	-1.7782568	0.00117483	0.01336768	Cofactors and Vitamins
2-palmitoleoyl-GPC (16:1)*	-2.0180099	0.00140022	0.01567227	Lipid
21-hydroxypregnenolone disulfate	1.56410003	0.00146197	0.01567227	Lipid
bilirubin degradation product, C17H18N2O4 (2)**	2.01402427	0.00146197	0.01567227	Phase 2 Conjugates/Bilirubin Catabolites
citrulline	-0.7462845	0.00146197	0.01567227	Amino Acid
17alpha-hydroxypregnanolone glucuronide	1.95249911	0.00147044	0.01567227	Lipid
9,10-DiHOME	3.00226757	0.00184026	0.01889638	Lipid
bilirubin degradation product, C16H18N2O5 (3)**	1.5534416	0.00184026	0.01889638	Phase 2 Conjugates/Bilirubin Catabolites
isovalerylcarnitine (C5)	-1.0616683	0.00184026	0.01889638	Amino Acid
1-methyladenosine	0.51124406	0.00230113	0.02201764	Nucleotide
fumarate	0.79667494	0.00230113	0.02201764	Energy
glycerol	-1.189832	0.00230113	0.02201764	Lipid
glycosyl ceramide (d18:2/24:1, d18:1/24:2)*	-1.0400781	0.00230113	0.02201764	Lipid
metabolonic lactone sulfate	-1.445402	0.00230113	0.02201764	Phase 2 Conjugates/Bilirubin Catabolites
17alpha-hydroxypregnenolone 3-sulfate	1.73253584	0.00247429	0.02340845	Lipid
beta-citrylglutamate	1.16861957	0.00253831	0.02348631	Amino Acid
hypoxanthine	-1.4161552	0.00253831	0.02348631	Nucleotide
1-linoleoyl-GPC (18:2)	-1.5771221	0.00285673	0.02405368	Lipid
cholesterol	-0.6613183	0.00285673	0.02405368	Lipid

glycosyl ceramide (d18:1/20:0, d16:1/22:0)*	-0.9231815	0.00285673	0.02405368	Lipid
lactosyl-N-nervonoyl-sphingosine (d18:1/24:1)*	-0.8347101	0.00285673	0.02405368	Lipid
linoleoylcarnitine (C18:2)*	-0.8998617	0.00285673	0.02405368	Lipid
oleoylcarnitine (C18:1)	-0.8568797	0.00285673	0.02405368	Lipid
spermidine	1.14128735	0.00285673	0.02405368	Amino Acid
sphingomyelin (d18:1/20:2, d18:2/20:1, d16:1/22:2)*	-0.8473137	0.00285673	0.02405368	Lipid
6-bromotryptophan	-1.1996868	0.00301354	0.02512279	Amino Acid
1-(1-enyl-oleoyl)-GPE (P-18:1)*	-1.1657606	0.00325668	0.02688361	Lipid
1-palmitoyl-2-dihomo-linolenoyl-GPC (16:0/20:3n3 or 6)*	-0.4404998	0.00352627	0.02749183	Lipid

Table S1: Significantly altered metabolites in VA-ECMO subjects compared with sick controls.

Metabolite	log2fc	p.value	p.adj	SUPER_PATHWAY
(N(1) + N(8))-acetylspermidine	1.94092026	9.44E-09	7.19E-07	Amino Acid
1-linoleoylglycerol (18:2)	4.96629825	9.44E-09	7.19E-07	Lipid
1-methyladenosine	1.30306065	9.44E-09	7.19E-07	Nucleotide
2-palmitoyl-GPC (16:0)*	-2.7908911	9.44E-09	7.19E-07	Lipid
aconitate [cis or trans]	3.12748051	9.44E-09	7.19E-07	Energy
asparagine	3.28292544	9.44E-09	7.19E-07	Amino Acid
biliverdin	3.76843249	9.44E-09	7.19E-07	Cofactors and Vitamins
citrate	1.62710024	9.44E-09	7.19E-07	Energy
gluconate	5.46898686	9.44E-09	7.19E-07	Xenobiotics
glutamine	2.19859823	9.44E-09	7.19E-07	Amino Acid
N-acetylputrescine	1.92290671	9.44E-09	7.19E-07	Amino Acid
21-hydroxypregnenolone monosulfate (2)	2.79541727	1.12E-08	7.84E-07	Lipid
1-palmitoyl-GPC (16:0)	-2.3684283	1.89E-08	9.89E-07	Lipid
1-stearoyl-GPC (18:0)	-2.9638256	1.89E-08	9.89E-07	Lipid
gamma-glutamylglutamine	1.57698541	1.89E-08	9.89E-07	Peptide
mannitol/sorbitol	5.20785055	1.89E-08	9.89E-07	Carbohydrate
3-(4-hydroxyphenyl)lactate (HPLA)	2.1819126	3.78E-08	1.67E-06	Amino Acid
retinol (vitamin A)	-1.6715626	3.78E-08	1.67E-06	Cofactors and Vitamins
sphingomyelin (d18:1/20:1, d18:2/20:0)*	-1.359147	3.78E-08	1.67E-06	Lipid
1-(1-enyl-stearoyl)-2-arachidonoyl-GPE (P-18:0/20:4)*	-1.7280924	1.13E-07	4.52E-06	Lipid
1-palmitoleoyl-GPC (16:1)*	-2.7389402	1.13E-07	4.52E-06	Lipid
1-arachidonylglycerol (20:4)	3.10456278	1.79E-07	6.26E-06	Lipid
1-linoleoyl-2-arachidonoyl-GPC (18:2/20:4n6)*	-2.0562068	1.79E-07	6.26E-06	Lipid
sphingomyelin (d18:2/24:1, d18:1/24:2)*	-0.9365682	1.79E-07	6.26E-06	Lipid
ibuprofen	4.9449632	1.93E-07	6.48E-06	Xenobiotics
adipoylcarnitine (C6-DC)	4.41348226	2.83E-07	8.18E-06	Lipid
phenyllactate (PLA)	1.80921296	2.83E-07	8.18E-06	Amino Acid
sphingomyelin (d18:2/21:0, d16:2/23:0)*	-1.6242243	2.83E-07	8.18E-06	Lipid
thyroxine	-1.7686621	2.83E-07	8.18E-06	Amino Acid
1-linoleoyl-GPC (18:2)	-2.0460042	4.25E-07	1.05E-05	Lipid
glucuronate	3.191302	4.25E-07	1.05E-05	Carbohydrate
sphingomyelin (d18:1/20:0, d16:1/22:0)*	-1.1440968	4.25E-07	1.05E-05	Lipid
sphingomyelin (d18:1/22:1, d18:2/22:0, d16:1/24:1)*	-1.219497	4.25E-07	1.05E-05	Lipid
sphingomyelin (d18:2/23:0, d18:1/23:1, d17:1/24:1)*	-1.5164167	4.25E-07	1.05E-05	Lipid
1-(1-enyl-palmitoyl)-2-linoleoyl-GPC (P-16:0/18:2)*	-1.24578	6.32E-07	1.32E-05	Lipid
1-(1-enyl-stearoyl)-GPE (P-18:0)*	-2.1241969	6.32E-07	1.32E-05	Lipid
1-oleoyl-GPC (18:1)	-1.9117779	6.32E-07	1.32E-05	Lipid
1-palmitoyl-2-dihomo-linolenoyl-GPC (16:0/20:3n3 or 6)*	-0.7991403	6.32E-07	1.32E-05	Lipid
sphingomyelin (d18:2/24:2)*	-1.2757754	6.32E-07	1.32E-05	Lipid
uridine	-2.4665618	6.32E-07	1.32E-05	Nucleotide
1-(1-enyl-palmitoyl)-GPE (P-16:0)*	-1.7676835	9.15E-07	1.57E-05	Lipid
1-arachidonoyl-GPC (20:4n6)*	-2.0321065	9.15E-07	1.57E-05	Lipid

1-dihomo-linolenylglycerol (20:3)	2.11073128	9.15E-07	1.57E-05	Lipid
2-pyrrolidinone	2.10812321	9.15E-07	1.57E-05	Amino Acid
catechol sulfate	-2.5908149	9.15E-07	1.57E-05	Xenobiotics
cystathionine	2.42137627	9.15E-07	1.57E-05	Amino Acid
lignoceroyl sphingomyelin (d18:1/24:0)	-1.4038438	9.15E-07	1.57E-05	Lipid
mannonate*	2.40274062	9.15E-07	1.57E-05	Xenobiotics
tricosanoyl sphingomyelin (d18:1/23:0)*	-1.4581444	9.15E-07	1.57E-05	Lipid
1-palmitoylglycerol (16:0)	2.33683735	1.30E-06	1.82E-05	Lipid
2-hydroxybutyrate/2-hydroxyisobutyrate	1.43322422	1.30E-06	1.82E-05	Amino Acid
3-carboxy-4-methyl-5-pentyl-2-furanpropionate (3-CMPFP)**	-1.6199723	1.30E-06	1.82E-05	Lipid
7-methylguanine	1.20861458	1.30E-06	1.82E-05	Nucleotide
behenoyl sphingomyelin (d18:1/22:0)*	-1.1717132	1.30E-06	1.82E-05	Lipid
glycerophosphorylcholine (GPC)	-2.256154	1.30E-06	1.82E-05	Lipid
myo-inositol	2.0693126	1.30E-06	1.82E-05	Lipid
N1-methylinosine	2.44892158	1.30E-06	1.82E-05	Nucleotide
sphingomyelin (d18:1/18:1, d18:2/18:0)	-0.8834873	1.30E-06	1.82E-05	Lipid
sphingomyelin (d18:1/19:0, d19:1/18:0)*	-1.3948747	1.30E-06	1.82E-05	Lipid
sphingomyelin (d18:1/25:0, d19:0/24:1, d20:1/23:0, d19:1/24:0)*	-1.5215894	1.30E-06	1.82E-05	Lipid
1-linolenoyl-GPC (18:3)*	-2.0409542	1.81E-06	2.27E-05	Lipid
1-oleoylglycerol (18:1)	3.60546915	1.81E-06	2.27E-05	Lipid
1-palmitoleoylglycerol (16:1)*	2.33496827	1.81E-06	2.27E-05	Lipid
sphingomyelin (d18:1/17:0, d17:1/18:0, d19:1/16:0)	-1.003535	1.81E-06	2.27E-05	Lipid
sphingomyelin (d18:1/21:0, d17:1/22:0, d16:1/23:0)*	-1.6246972	1.81E-06	2.27E-05	Lipid
sphingomyelin (d18:1/24:1, d18:2/24:0)*	-0.807047	1.81E-06	2.27E-05	Lipid
sphingomyelin (d18:2/16:0, d18:1/16:1)*	-0.791467	1.81E-06	2.27E-05	Lipid
1-palmitoyl-2-oleoyl-GPE (16:0/18:1)	2.03689832	2.50E-06	2.99E-05	Lipid
2,6-dihydroxybenzoic acid	-2.4800633	2.50E-06	2.99E-05	Xenobiotics
sphingomyelin (d18:2/23:1)*	-1.2472147	2.50E-06	2.99E-05	Lipid
3-hydroxyhippurate	-4.2285536	3.39E-06	4.00E-05	Xenobiotics
1-palmitoyl-2-stearoyl-GPC (16:0/18:0)	-0.6731876	4.55E-06	4.89E-05	Lipid
1-stearoyl-2-linoleoyl-GPC (18:0/18:2)*	-0.4450847	4.55E-06	4.89E-05	Lipid
alpha-hydroxyisovalerate	1.2846894	4.55E-06	4.89E-05	Amino Acid
alpha-tocopherol	-0.9036188	4.55E-06	4.89E-05	Cofactors and Vitamins
bilirubin degradation product, C17H18N2O4 (2)**	2.73132809	4.55E-06	4.89E-05	Phase 2 Conjugates/Bilirubin Catabolites
hydantoin-5-propionate	2.54520903	4.55E-06	4.89E-05	Amino Acid
sphingomyelin (d18:0/20:0, d16:0/22:0)*	-1.5467856	4.55E-06	4.89E-05	Lipid
1-(1-enyl-palmitoyl)-2-arachidonoyl-GPC (P-16:0/20:4)*	-1.0035937	6.03E-06	5.95E-05	Lipid
1-myristoyl-2-arachidonoyl-GPC (14:0/20:4)*	-1.8217432	6.03E-06	5.95E-05	Lipid
1,2-dilinoleoyl-GPC (18:2/18:2)	-1.5253247	6.03E-06	5.95E-05	Lipid
2-linoleoylglycerol (18:2)	3.65051166	6.03E-06	5.95E-05	Lipid
citrulline	-0.9728782	6.03E-06	5.95E-05	Amino Acid
palmitoyl sphingomyelin (d18:1/16:0)	-0.4219225	6.03E-06	5.95E-05	Lipid
tetrahydrocortisol glucuronide	2.24646374	6.03E-06	5.95E-05	Lipid

1,2,3-benzenetriol sulfate (2)	-6.5840393	6.72E-06	6.47E-05	Xenobiotics
2-palmitoleoyl-GPC (16:1)*	-3.1243654	6.72E-06	6.47E-05	Lipid
alpha-ketobutyrate	2.38912628	7.19E-06	6.84E-05	Amino Acid
6-bromotryptophan	-2.4215367	7.32E-06	6.84E-05	Amino Acid
2-hydroxy-4-(methylthio)butanoic acid	1.73344475	7.34E-06	6.84E-05	Amino Acid
bilirubin degradation product, C17H18N2O4 (3)**	3.05385601	7.93E-06	7.22E-05	Phase 2 Conjugates/Bilirubin Catabolites
sphingomyelin (d18:2/18:1)*	-1.4886937	7.93E-06	7.22E-05	Lipid
1-(1-enyl-palmitoyl)-GPC (P-16:0)*	-3.3499694	8.09E-06	7.29E-05	Lipid
1-(1-enyl-palmitoyl)-2-arachidonoyl-GPE (P-16:0/20:4)*	-1.0571289	1.03E-05	9.09E-05	Lipid
kynurenine	0.91428138	1.03E-05	9.09E-05	Amino Acid
4-methoxyphenol sulfate	3.45956708	1.10E-05	9.63E-05	Amino Acid
trigonelline (N'-methylnicotinate)	-2.8586496	1.33E-05	0.00011496	Cofactors and Vitamins
1-(1-enyl-stearoyl)-2-linoleoyl-GPE (P-18:0/18:2)*	-1.0857981	1.70E-05	0.0001398	Lipid
1-oleoyl-2-docosahexaenoyl-GPC (18:1/22:6)*	-1.1712891	1.70E-05	0.0001398	Lipid
eicosenedioate (C20:1-DC)*	-1.7595742	1.70E-05	0.0001398	Lipid
orotate	2.16326475	1.70E-05	0.0001398	Nucleotide
sphingomyelin (d17:1/16:0, d18:1/15:0, d16:1/17:0)*	-0.8125525	1.70E-05	0.0001398	Lipid
1-linolenoylglycerol (18:3)	4.19575239	1.84E-05	0.00014965	Lipid
myristoyl dihydrosphingomyelin (d18:0/14:0)*	-1.0781206	2.16E-05	0.00017422	Lipid
1-(1-enyl-oleoyl)-GPE (P-18:1)*	-2.8106555	2.28E-05	0.00018202	Lipid
uracil	-3.6107751	2.67E-05	0.00021082	Nucleotide
phosphate	0.63119989	2.73E-05	0.00021354	Energy
4-acetamidobutanoate	2.2035602	3.32E-05	0.00025774	Amino Acid
pyruvate	1.82273416	3.42E-05	0.00026034	Carbohydrate
stearoyl sphingomyelin (d18:1/18:0)	-0.6887264	3.42E-05	0.00026034	Lipid
3-(N-acetyl-cystein-S-yl) acetaminophen	4.03779575	3.56E-05	0.00026851	Xenobiotics
glycosyl-N-behenoyl-sphingadienine (d18:2/22:0)*	-1.6434864	3.84E-05	0.00028735	Lipid
imidazole propionate	4.8748209	4.25E-05	0.00031551	Amino Acid
naproxen	0.61670733	4.41E-05	0.00032438	Xenobiotics
isoursodeoxycholate	-3.5467388	4.73E-05	0.00034446	Lipid
4-acetamidophenylglucuronide	1.89385575	4.95E-05	0.00035762	Xenobiotics
1-stearoyl-2-arachidonoyl-GPC (18:0/20:4)	-0.4314956	5.27E-05	0.00036498	Lipid
2-ketocaprylate	-1.2804088	5.27E-05	0.00036498	Amino Acid
behenoyl dihydrosphingomyelin (d18:0/22:0)*	-1.3281869	5.27E-05	0.00036498	Lipid
carotene diol (3)	-1.4971696	5.27E-05	0.00036498	Cofactors and Vitamins
linoleoylcarnitine (C18:2)*	1.17200648	5.27E-05	0.00036498	Lipid
N-acetylglucosamine/N-acetylgalactosamine	1.74825938	5.90E-05	0.0004051	Carbohydrate
1-stearoyl-2-arachidonoyl-GPI (18:0/20:4)	-0.8922254	6.49E-05	0.0004215	Lipid
gamma-glutamylcitrulline*	-1.0837334	6.49E-05	0.0004215	Peptide
glycosyl ceramide (d18:1/20:0, d16:1/22:0)*	-0.9675581	6.49E-05	0.0004215	Lipid
N-acetyl-isoputreanine	1.40521056	6.49E-05	0.0004215	Amino Acid
perfluorooctanesulfonate (PFOS)	-3.2933589	6.49E-05	0.0004215	Xenobiotics
sphingomyelin (d18:2/14:0, d18:1/14:1)*	-1.1473115	6.49E-05	0.0004215	Lipid

sphingosine	1.67923728	6.49E-05	0.0004215	Lipid
leucylalanine	1.62513283	7.06E-05	0.00045519	Peptide
2-oleoylglycerol (18:1)	3.3884291	7.75E-05	0.00048631	Lipid
4-acetaminophen sulfate	3.41627237	7.82E-05	0.00048631	Xenobiotics
1-(1-enyl-palmitoyl)-2-linoleoyl-GPE (P-16:0/18:2)*	-1.0156982	7.95E-05	0.00048631	Lipid
1-stearoyl-2-oleoyl-GPE (18:0/18:1)	1.81646439	7.95E-05	0.00048631	Lipid
5alpha-pregnan-3beta,20alpha-diol disulfate	2.03951093	7.95E-05	0.00048631	Lipid
indolepropionate	-2.3903673	7.95E-05	0.00048631	Amino Acid
N-acetyl-beta-alanine	0.89687082	7.95E-05	0.00048631	Nucleotide
3-methoxycatechol sulfate (1)	-4.5751219	8.83E-05	0.00053629	Xenobiotics
1-pentadecanoylglycerol (15:0)	2.28035846	9.34E-05	0.00056307	Lipid
dimethyl sulfone	-1.2690138	9.69E-05	0.00056794	Xenobiotics
N-acetyl-2-aminoadipate	1.15892607	9.69E-05	0.00056794	Amino Acid
N,N-dimethyl-pro-pro	1.3603345	9.69E-05	0.00056794	Peptide
phenylalanine	0.48387718	9.69E-05	0.00056794	Amino Acid
sphingomyelin (d17:2/16:0, d18:2/15:0)*	-1.3738516	0.0001027	0.00059766	Lipid
1-palmitoyl-2-arachidonoyl-GPC (16:0/20:4n6)	-0.382108	0.00011762	0.00065712	Lipid
beta-cryptoxanthin	-1.6862308	0.00011762	0.00065712	Cofactors and Vitamins
carotene diol (1)	-1.615878	0.00011762	0.00065712	Cofactors and Vitamins
gamma-glutamylphenylalanine	0.96171066	0.00011762	0.00065712	Peptide
sphingomyelin (d18:1/22:2, d18:2/22:1, d16:1/24:2)*	-0.9684397	0.00011762	0.00065712	Lipid
taurocholenate sulfate*	3.13775469	0.00011762	0.00065712	Lipid
sphingomyelin (d17:1/14:0, d16:1/15:0)*	-1.603997	0.00013484	0.00074516	Lipid
orotidine	3.86797844	0.00013516	0.00074516	Nucleotide
cortisol	1.13103943	0.00014209	0.00075839	Lipid
kynurenate	2.85620873	0.00014209	0.00075839	Amino Acid
linoleoyl ethanolamide	1.66744547	0.00014209	0.00075839	Lipid
mannose	0.92822992	0.00014209	0.00075839	Carbohydrate
pregnenolone sulfate	2.0108246	0.00014209	0.00075839	Lipid
butyrate/isobutyrate (4:0)	0.68174309	0.00017096	0.00090676	Lipid
2-methoxyacetaminophen sulfate*	2.58493268	0.00017315	0.00091258	Xenobiotics
1-palmitoyl-2-oleoyl-GPC (16:0/18:1)	0.35703618	0.0002445	0.00124935	Lipid
2-hydroxy-3-methylvalerate	1.41431802	0.0002445	0.00124935	Amino Acid
4-allylphenol sulfate	-3.8093707	0.0002445	0.00124935	Xenobiotics
alpha-hydroxyisocaproate	1.57084805	0.0002445	0.00124935	Amino Acid
homostachydrine*	-2.8606821	0.0002445	0.00124935	Xenobiotics
cysteinylglycine	1.76274497	0.00024674	0.00125316	Amino Acid
17alpha-hydroxypregnanolone glucuronide	1.81227854	0.0002557	0.00129082	Lipid
perfluorohexanesulfonate (PFHxS)	-2.6613975	0.00026205	0.00131497	Xenobiotics
glycosyl-N-tricosanoyl-sphingadienine (d18:2/23:0)*	-2.2210231	0.00027286	0.00136107	Lipid
1-palmitoyl-2-linoleoyl-GPE (16:0/18:2)	1.54456658	0.00029075	0.00143322	Lipid
21-hydroxypregnenolone disulfate	2.03621516	0.00029075	0.00143322	Lipid
glycodeoxycholate 3-sulfate	3.09265802	0.00029821	0.00145291	Lipid

o-cresol sulfate	1.03691406	0.00029821	0.00145291	Xenobiotics
indoleacetoylcarnitine*	-2.0380737	0.0003118	0.00151033	Amino Acid
malonate	1.5947174	0.00031986	0.00154046	Lipid
GlcNAc sulfate conjugate of C21H34O2 steroid**	3.10033847	0.00032623	0.00156215	Phase 2 Conjugates/Bilirubin Catabolites
1,2-dipalmitoyl-GPE (16:0/16:0)*	1.04836132	0.00032818	0.00156259	Lipid
N-acetyltryptophan	8.01399928	0.000339	0.00158646	Amino Acid
2-methoxyacetaminophen glucuronide*	0.65996033	0.00033962	0.00158646	Xenobiotics
oxypurinol	1.71067484	0.00033962	0.00158646	Xenobiotics
palmitoyl dihydrosphingomyelin (d18:0/16:0)*	-0.5246842	0.00034455	0.00158646	Lipid
pregnenetriol disulfate*	2.57345262	0.00034455	0.00158646	Lipid
sphingomyelin (d18:1/14:0, d16:1/16:0)*	-0.7517713	0.00034455	0.00158646	Lipid
alpha-ketoglutaramate*	1.24608342	0.00040687	0.00186316	Amino Acid
lactosyl-N-behenoyl-sphingosine (d18:1/22:0)*	-1.1188473	0.00046464	0.00207961	Lipid
histidine betaine (hercynine)*	-1.9510264	0.00046495	0.00207961	Xenobiotics
1,5-anhydroglucitol (1,5-AG)	-1.3046085	0.00047896	0.00207961	Carbohydrate
bilirubin degradation product, C17H18N2O4 (1)**	2.5272085	0.00047896	0.00207961	Phase 2 Conjugates/Bilirubin Catabolites
glycerol	1.58276869	0.00047896	0.00207961	Lipid
guanidinoacetate	-1.1905656	0.00047896	0.00207961	Amino Acid
hypotaurine	1.53969777	0.00047896	0.00207961	Amino Acid
imidazole lactate	1.40741869	0.00047896	0.00207961	Amino Acid
N,N,N-trimethyl-alanylproline betaine (TMAP)	1.88103337	0.00047896	0.00207961	Amino Acid
S-methylcysteine sulfoxide	-1.5522034	0.00047896	0.00207961	Amino Acid
1-myristoylglycerol (14:0)	1.57687907	0.00049489	0.0021377	Lipid
formiminoglutamate	3.15331035	0.00055914	0.00236641	Amino Acid
1-palmitoyl-GPE (16:0)	-0.9765437	0.00056195	0.00236641	Lipid
2-hydroxyoctanoate	-0.7556608	0.00056195	0.00236641	Lipid
fumarate	0.97697652	0.00056195	0.00236641	Energy
taurine	-0.714588	0.00056195	0.00236641	Amino Acid
chenodeoxycholate	-3.8919663	0.00056594	0.00237128	Lipid
pregnanolone/allopregnanolone sulfate	2.29631558	0.00062671	0.00261286	Lipid
ribitol	1.29126807	0.00063232	0.00261525	Carbohydrate
beta-citrylglutamate	1.38611922	0.00063353	0.00261525	Amino Acid
2-keto-3-deoxy-gluconate	1.00478209	0.00065738	0.00266126	Xenobiotics
arginine	-0.7613533	0.00065738	0.00266126	Amino Acid
glycerate	-1.1312887	0.00065738	0.00266126	Carbohydrate
N-acetyltaurine	2.19790837	0.00065738	0.00266126	Amino Acid
androstenediol (3beta,17beta) monosulfate (2)	-1.8436754	0.00071567	0.00288333	Lipid
riboflavin (vitamin B2)	4.36726346	0.00075305	0.00301631	Cofactors and Vitamins
1-dihomo-linoleoylglycerol (20:2)	1.92209779	0.00075724	0.00301631	Lipid
eugenol sulfate	-4.0474006	0.00076339	0.00301631	Xenobiotics
2-aminophenol sulfate	-1.9793416	0.00076667	0.00301631	Xenobiotics
carotene diol (2)	-1.2685938	0.00076667	0.00301631	Cofactors and Vitamins
4-acetamidophenol	2.38950904	0.0007823	0.00306341	Xenobiotics

glutamate	-1.0512937	0.00089165	0.00345927	Amino Acid
theobromine	-2.0576567	0.00089165	0.00345927	Xenobiotics
bilirubin degradation product, C16H18N2O5 (3)**	1.60475599	0.00090008	0.00347589	Phase 2 Conjugates/Bilirubin Catabolites
pentose acid*	-2.4536267	0.00090552	0.00348086	Phase 2 Conjugates/Bilirubin Catabolites
N-acetylglutamate	0.80810007	0.00101383	0.00381732	Amino Acid
hypoxanthine	-3.0226925	0.00102346	0.00381732	Nucleotide
5alpha-pregnan-diol disulfate	4.61283296	0.00103376	0.00381732	Lipid
2S,3R-dihydroxybutyrate	1.66929522	0.00103405	0.00381732	Lipid
cholesterol	-0.581069	0.00103405	0.00381732	Lipid
cysteine s-sulfate	1.44150802	0.00103405	0.00381732	Amino Acid
homoarginine	-1.1433188	0.00103405	0.00381732	Amino Acid
oxindolylalanine	-1.0301966	0.00103405	0.00381732	Amino Acid
threonate	-1.1353329	0.00103405	0.00381732	Cofactors and Vitamins
bilirubin degradation product, C16H18N2O5 (1)**	1.57485981	0.0010469	0.00384781	Phase 2 Conjugates/Bilirubin Catabolites
docosadienoate (22:2n6)	1.61303938	0.00119605	0.0043578	Lipid
N-acetylarginine	-0.8494499	0.00119605	0.0043578	Amino Acid
inosine	-3.5661233	0.00129616	0.00470207	Nucleotide
L-urobilin	4.85308694	0.00132352	0.00478063	Cofactors and Vitamins
3-decenoylcarnitine	-1.1713725	0.00136078	0.00481756	Lipid
cytosine	-2.1559306	0.00136078	0.00481756	Nucleotide
2-aminooctanoate	-1.0259082	0.00137973	0.00481756	Lipid
2-hydroxydecanoate	-1.148343	0.00137973	0.00481756	Lipid
4-hydroxychlorothalonil	-0.96387	0.00137973	0.00481756	Xenobiotics
glycerophosphoethanolamine	-0.6814129	0.00137973	0.00481756	Lipid
serine	-0.5354022	0.00137973	0.00481756	Amino Acid
taurochenodeoxycholate	3.87771194	0.00137973	0.00481756	Lipid
taurodeoxycholic acid 3-sulfate	5.17695096	0.00142772	0.00496445	Lipid
picolinate	1.12407693	0.00145266	0.00501796	Amino Acid
glycochenodeoxycholate 3-sulfate	3.09655788	0.00145509	0.00501796	Lipid
pantoprazole	2.87602961	0.00156058	0.00534325	Xenobiotics
benzoate	-1.4017133	0.00158767	0.00534325	Xenobiotics
bilirubin degradation product, C17H20N2O5 (2)**	1.44716221	0.00158767	0.00534325	Phase 2 Conjugates/Bilirubin Catabolites
methionine	0.61741206	0.00158767	0.00534325	Amino Acid
N6,N6,N6-trimethyllysine	1.29535527	0.00158767	0.00534325	Amino Acid
palmitoyl-linoleoyl-glycerol (16:0/18:2) [2]*	1.69777717	0.00158767	0.00534325	Lipid
nervonate (24:1n9)*	1.31272802	0.00159466	0.00534531	Lipid
N2,N2-dimethylguanosine	1.44029158	0.00163181	0.00544805	Nucleotide
2-hydroxyacetaminophen sulfate*	1.95345568	0.00165598	0.00550679	Xenobiotics
17alpha-hydroxypregnenolone 3-sulfate	1.69402049	0.00179168	0.0059345	Lipid
1-stearoyl-GPE (18:0)	-0.8391122	0.00182237	0.00594221	Lipid
N-palmitoyl-sphinganine (d18:0/16:0)	0.86214867	0.00182237	0.00594221	Lipid
pregnen-diol disulfate*	2.09871024	0.00182237	0.00594221	Lipid
succinylcarnitine (C4-DC)	0.71361323	0.00182237	0.00594221	Energy

glycosyl ceramide (d18:2/24:1, d18:1/24:2)*	-0.7739166	0.00208682	0.0067002	Lipid
linolenoylcarnitine (C18:3)*	-0.930119	0.00208682	0.0067002	Lipid
N-acetylalanine	0.57378052	0.00208682	0.0067002	Amino Acid
N4-acetylcytidine	1.3303706	0.00208682	0.0067002	Nucleotide
valerate (5:0)	-1.2860653	0.00214998	0.00687664	Lipid
2-palmitoylglycerol (16:0)	1.56041002	0.00224748	0.00716117	Lipid
desmethylnaproxen sulfate	0.94490977	0.00235965	0.00749011	Xenobiotics
2,4-di-tert-butylphenol	-1.0521952	0.00238394	0.00751032	Xenobiotics
sphingosine 1-phosphate	-0.7553979	0.00238394	0.00751032	Lipid
glycoursodeoxycholic acid sulfate (1)	2.28586314	0.00255056	0.00794991	Lipid
sphingadienine	-3.3523233	0.00255056	0.00794991	Lipid
4-hydroxyphenylacetate	2.16591389	0.00255194	0.00794991	Amino Acid
estrone 3-sulfate	4.11138976	0.00260968	0.00809968	Lipid
cortolone glucuronide (1)	1.07925463	0.0027173	0.00834103	Lipid
fructosyllysine	0.96790441	0.0027173	0.00834103	Amino Acid
octadecenedioylcarnitine (C18:1-DC)*	2.67683909	0.0027173	0.00834103	Lipid
3-(methylthio)acetaminophen sulfate*	3.0154408	0.00272933	0.00834737	Xenobiotics
bilirubin (E,Z or Z,E)*	-1.6104328	0.0028023	0.00853936	Cofactors and Vitamins
taurochenodeoxycholic acid 3-sulfate	4.47471571	0.00282322	0.00857196	Lipid
sebacate (C10-DC)	-1.5714538	0.00284565	0.0085906	Lipid
homocitrulline	1.82463737	0.00284987	0.0085906	Amino Acid
1-palmitoleoyl-2-linolenoyl-GPC (16:1/18:3)*	-0.9689785	0.00299579	0.00896598	Lipid
N-acetylhistidine	0.98568675	0.00299579	0.00896598	Amino Acid
1-palmitoyl-2-stearoyl-GPE (16:0/18:0)*	0.92281634	0.00307821	0.00911846	Lipid
2-stearoyl-GPE (18:0)*	-1.2454354	0.00309027	0.00911846	Lipid
oxalate (ethanedioate)	-1.4054662	0.00309027	0.00911846	Cofactors and Vitamins

Table S2. Significantly altered metabolites in VA-ECMO subjects compared with healthy controls

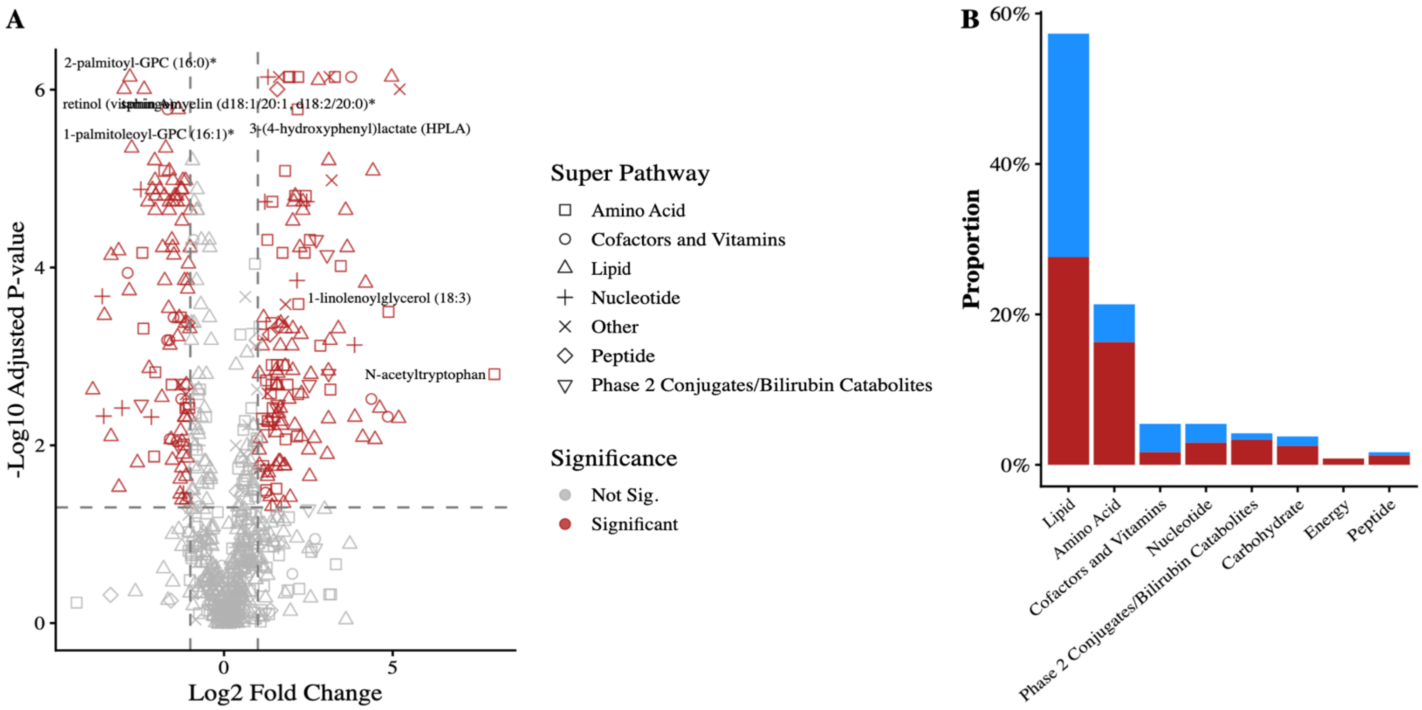

Figure S1. VA-ECMO vs healthy controls. Volcano plot comparing VA-ECMO subjects to healthy controls (A). Proportion of altered metabolite classes (B).

Metabolite	log2fc	p.value	p.adj	SUPER_PATHWAY
1-(1-enyl-palmitoyl)-2-arachidonoyl-GPC (P-16:0/20:4)*	-1.3201436	4.33E-07	0.00036442	Lipid
1-(1-enyl-palmitoyl)-2-linoleoyl-GPC (P-16:0/18:2)*	-1.4003492	3.72E-06	0.00156764	Lipid
taurine	-1.0172227	8.58E-06	0.00233167	Amino Acid
1-stearoyl-2-arachidonoyl-GPC (18:0/20:4)	-0.5523873	1.23E-05	0.00233167	Lipid
1-(1-enyl-stearoyl)-2-arachidonoyl-GPE (P-18:0/20:4)*	-1.6770717	1.38E-05	0.00233167	Lipid
1-palmitoleoylglycerol (16:1)*	1.77540089	1.68E-05	0.00235602	Lipid
sphingomyelin (d18:1/20:1, d18:2/20:0)*	-0.7178045	3.03E-05	0.00365029	Lipid
sphinganine	0.80040843	6.12E-05	0.00643879	Lipid
1-(1-enyl-palmitoyl)-2-oleoyl-GPC (P-16:0/18:1)*	-0.8914633	6.95E-05	0.00650461	Lipid
1-dihomo-linolenylglycerol (20:3)	1.26406547	7.85E-05	0.00661352	Lipid
1-dihomo-linoleoylglycerol (20:2)	2.51475578	0.00011	0.00842006	Lipid
citrate	-0.1858486	0.00016717	0.00998485	Energy
2-palmitoylglycerol (16:0)	1.75173387	0.00017285	0.00998485	Lipid
2-oleoylglycerol (18:1)	2.75517225	0.00017343	0.00998485	Lipid
sphingomyelin (d18:2/24:2)*	-0.5795585	0.00018827	0.00998485	Lipid
glycosyl-N-behenoyl-sphingadienine (d18:2/22:0)*	-1.3798784	0.00018974	0.00998485	Lipid
1-palmitoylglycerol (16:0)	1.95215286	0.00024226	0.01199913	Lipid
sphingosine	1.00646923	0.00027874	0.01303868	Lipid
docosadienoate (22:2n6)	0.91495258	0.00032735	0.01417235	Lipid
sphingomyelin (d18:1/25:0, d19:0/24:1, d20:1/23:0, d19:1/24:0)*	-1.2931436	0.00035404	0.01417235	Lipid
arachidonoylcarnitine (C20:4)	-1.1897377	0.0003569	0.01417235	Lipid
palmitoyl-linoleoyl-glycerol (16:0/18:2) [2]*	1.36727268	0.0003703	0.01417235	Lipid
6-bromotryptophan	-1.0300118	0.00044847	0.01641806	Amino Acid
1-oleoylglycerol (18:1)	2.45325725	0.00049989	0.01753792	Lipid
oleoyl-linoleoyl-glycerol (18:1/18:2) [2]	0.8265509	0.00053561	0.01780275	Lipid
1-palmitoyl-2-linoleoyl-GPE (16:0/18:2)	1.3535038	0.00056768	0.01780275	Lipid
trans-urocanate	-2.3573151	0.00057087	0.01780275	Amino Acid
glycosyl ceramide (d18:1/20:0, d16:1/22:0)*	-1.1210476	0.00059406	0.0178642	Lipid
1-palmitoyl-2-linoleoyl-GPC (16:0/18:2)	0.28828814	0.00063873	0.01832641	Lipid
pipecolate	1.45475909	0.00065296	0.01832641	Amino Acid
guanidinoacetate	-1.6041658	0.00072789	0.01977047	Amino Acid
1-(1-enyl-oleoyl)-GPE (P-18:1)*	-1.116805	0.00078284	0.02059835	Lipid
dimethyl sulfone	-0.8592292	0.00093176	0.0229625	Xenobiotics
sphingomyelin (d18:2/23:0, d18:1/23:1, d17:1/24:1)*	-0.7604461	0.000933	0.0229625	Lipid
gamma-glutamylcitrulline*	-1.1926893	0.0009545	0.0229625	Peptide
stearoylcarnitine (C18)	-0.7281045	0.00105461	0.02466621	Lipid
pentose acid*	-4.0404059	0.00114069	0.0259585	Phase 2 Conjugates/Bilirubin Catabolites

Table S3. Significantly altered metabolites in VV-ECMO subjects compared with sick controls

Metabolite	log2fc	p.value	p.adj	SUPER_PATHWAY
1-(1-enyl-palmitoyl)-GPC (P-16:0)*	-2.1207961	1.82E-15	1.53E-12	Lipid
6-bromotryptophan	-2.2518617	5.21E-15	2.19E-12	Amino Acid
citrate	1.747779	2.10E-14	5.88E-12	Energy
1-(1-enyl-palmitoyl)-2-linoleoyl-GPC (P-16:0/18:2)*	-1.900866	2.27E-13	4.77E-11	Lipid
guanidinoacetate	-2.4851927	4.53E-13	7.62E-11	Amino Acid
1-stearoyl-GPC (18:0)	-1.6591127	2.43E-12	3.40E-10	Lipid
sphingomyelin (d18:1/20:1, d18:2/20:0)*	-1.0081061	2.88E-12	3.47E-10	Lipid
glycosyl-N-behenoyl-sphingadienine (d18:2/22:0)*	-1.7878502	1.11E-11	1.17E-09	Lipid
1-(1-enyl-palmitoyl)-2-oleoyl-GPC (P-16:0/18:1)*	-1.111797	1.34E-11	1.23E-09	Lipid
1-(1-enyl-palmitoyl)-2-arachidonoyl-GPC (P-16:0/20:4)*	-1.3787465	1.47E-11	1.23E-09	Lipid
uracil	-3.1340898	1.66E-11	1.27E-09	Nucleotide
1-(1-enyl-stearoyl)-2-arachidonoyl-GPE (P-18:0/20:4)*	-2.2090156	1.99E-11	1.39E-09	Lipid
1-(1-enyl-stearoyl)-GPE (P-18:0)*	-1.9230664	2.71E-11	1.75E-09	Lipid
citrulline	-0.9157163	6.77E-11	4.07E-09	Amino Acid
1-(1-enyl-oleoyl)-GPE (P-18:1)*	-2.7616999	2.04E-10	1.15E-08	Lipid
1-(1-enyl-palmitoyl)-GPE (P-16:0)*	-1.7042264	3.06E-10	1.61E-08	Lipid
glutamine	2.26585142	3.30E-10	1.63E-08	Amino Acid
3-carboxy-4-methyl-5-pentyl-2-furanpropionate (3-CMPFP)**	-2.4355029	4.93E-10	2.30E-08	Lipid
sphingomyelin (d18:1/25:0, d19:0/24:1, d20:1/23:0, d19:1/24:0)*	-1.5333445	5.48E-10	2.43E-08	Lipid
sphingomyelin (d18:2/21:0, d16:2/23:0)*	-1.2827066	8.24E-10	3.46E-08	Lipid
S-methylcysteine	-1.2408893	1.64E-09	6.55E-08	Amino Acid
1-palmitoleoyl-GPC (16:1)*	-1.436502	2.04E-09	7.80E-08	Lipid
2-hydroxyoctanoate	-1.1994627	2.34E-09	8.55E-08	Lipid
sphingomyelin (d17:1/14:0, d16:1/15:0)*	-1.6942392	2.53E-09	8.83E-08	Lipid
1-(1-enyl-palmitoyl)-2-arachidonoyl-GPE (P-16:0/20:4)*	-1.335933	2.62E-09	8.83E-08	Lipid
sphingomyelin (d18:1/21:0, d17:1/22:0, d16:1/23:0)*	-1.4963085	4.25E-09	1.38E-07	Lipid
sphingomyelin (d18:2/23:0, d18:1/23:1, d17:1/24:1)*	-1.2120956	6.31E-09	1.97E-07	Lipid
N-acetylglucosamine/N-acetylgalactosamine	1.62571278	7.56E-09	2.27E-07	Carbohydrate
sphingomyelin (d18:1/22:1, d18:2/22:0, d16:1/24:1)*	-0.8161559	9.76E-09	2.83E-07	Lipid
1-(1-enyl-palmitoyl)-2-linoleoyl-GPE (P-16:0/18:2)*	-1.6853197	1.09E-08	3.05E-07	Lipid
catechol sulfate	-4.0990865	1.16E-08	3.14E-07	Xenobiotics
asparagine	3.11072838	1.55E-08	4.09E-07	Amino Acid
1-linoleoyl-2-arachidonoyl-GPC (18:2/20:4n6)*	-1.334248	2.81E-08	7.16E-07	Lipid
sphingomyelin (d18:2/14:0, d18:1/14:1)*	-1.1471338	3.84E-08	9.51E-07	Lipid
1-oleoyl-2-docosahexaenoyl-GPC (18:1/22:6)*	-1.0677285	4.88E-08	1.17E-06	Lipid
glycosyl ceramide (d18:1/20:0, d16:1/22:0)*	-1.1654241	5.37E-08	1.25E-06	Lipid
2-palmitoleoyl-GPC (16:1)*	-2.0047297	6.45E-08	1.47E-06	Lipid
glycerate	-1.2320048	6.80E-08	1.50E-06	Carbohydrate
sulfate of piperine metabolite C18H21NO3 (3)*	-3.3291625	7.12E-08	1.54E-06	Xenobiotics
1-oleoyl-GPC (18:1)	-0.9901649	8.94E-08	1.88E-06	Lipid
2-ketocaprylate	-1.8192381	1.04E-07	2.13E-06	Amino Acid
2-hydroxyphytanate*	-2.0179674	1.83E-07	3.66E-06	Lipid

4-allylphenol sulfate	-5.7769137	1.88E-07	3.68E-06	Xenobiotics
carotene diol (1)	-1.821679	1.92E-07	3.68E-06	Cofactors and Vitamins
1-(1-enyl-stearoyl)-2-linoleoyl-GPE (P-18:0/18:2)*	-1.2186338	2.12E-07	3.97E-06	Lipid
2-aminooctanoate	-1.5593509	2.39E-07	4.28E-06	Lipid
sphingomyelin (d18:1/19:0, d19:1/18:0)*	-1.0307405	2.42E-07	4.28E-06	Lipid
sulfate of piperine metabolite C18H21NO3 (1)*	-2.9391123	2.49E-07	4.28E-06	Xenobiotics
indolepropionate	-4.2768253	2.54E-07	4.28E-06	Amino Acid
uridine	-1.3649341	2.54E-07	4.28E-06	Nucleotide
1-palmitoyl-GPC (16:0)	-0.9772656	2.95E-07	4.84E-06	Lipid
dimethyl sulfone	-1.3426925	2.99E-07	4.84E-06	Xenobiotics
N-acetyl-2-aminoadipate	1.56554118	3.25E-07	5.16E-06	Amino Acid
glycosyl-N-tricosanoyl-sphingadienine (d18:2/23:0)*	-2.228021	3.42E-07	5.32E-06	Lipid
adenine	-1.0774024	3.72E-07	5.69E-06	Nucleotide
2,6-dihydroxybenzoic acid	-5.0920901	3.81E-07	5.72E-06	Xenobiotics
gamma-glutamylglutamine	1.66040462	3.90E-07	5.76E-06	Peptide
tricosanoyl sphingomyelin (d18:1/23:0)*	-1.0215072	6.41E-07	9.15E-06	Lipid
trans-urocanate	-2.3493728	6.42E-07	9.15E-06	Amino Acid
aconitate [cis or trans]	1.80448384	7.07E-07	9.91E-06	Energy
1-dihomo-linolenylglycerol (20:3)	3.62290321	7.67E-07	1.06E-05	Lipid
1-palmitoyl-2-linoleoyl-GPC (16:0/18:2)	0.39749253	9.68E-07	1.31E-05	Lipid
mannose	1.86357952	9.79E-07	1.31E-05	Carbohydrate
carotene diol (2)	-1.5743459	1.06E-06	1.39E-05	Cofactors and Vitamins
carotene diol (3)	-1.7324523	1.14E-06	1.47E-05	Cofactors and Vitamins
1-methyladenosine	1.0655264	1.23E-06	1.55E-05	Nucleotide
branched chain 14:0 dicarboxylic acid**	-4.0572861	1.24E-06	1.55E-05	Lipid
alpha-ketobutyrate	2.59571557	1.28E-06	1.59E-05	Amino Acid
sphingosine	2.29149923	1.35E-06	1.63E-05	Lipid
2-aminophenol sulfate	-4.3483606	1.36E-06	1.63E-05	Xenobiotics
1-palmitoleoylglycerol (16:1)*	3.51773879	1.46E-06	1.73E-05	Lipid
sphingomyelin (d18:2/18:1)*	-1.2544605	1.62E-06	1.89E-05	Lipid
sulfate of piperine metabolite C16H19NO3 (2)*	-2.998345	1.95E-06	2.25E-05	Xenobiotics
glycerophosphorylcholine (GPC)	-1.5369144	2.22E-06	2.52E-05	Lipid
5-methyluridine (ribothymidine)	-1.1211352	2.82E-06	3.16E-05	Nucleotide
oxindolylalanine	-1.133336	3.15E-06	3.49E-05	Amino Acid
docosadienoate (22:2n6)	1.76072108	3.58E-06	3.91E-05	Lipid
N,N,N-trimethyl-5-aminovalerate	-0.8892666	3.67E-06	3.96E-05	Amino Acid
perfluorooctanesulfonate (PFOS)	-3.0093667	4.62E-06	4.92E-05	Xenobiotics
1,2-dilinoleoyl-GPC (18:2/18:2)	-0.9611211	4.83E-06	5.08E-05	Lipid
homoarginine	-1.2561268	5.09E-06	5.29E-05	Amino Acid
malonate	1.57312886	5.38E-06	5.51E-05	Lipid
2-hydroxy-3-methylvalerate	2.3666394	5.96E-06	6.03E-05	Amino Acid
sphingomyelin (d18:1/14:0, d16:1/16:0)*	-0.6610099	6.02E-06	6.03E-05	Lipid
thyroxine	-0.7936657	6.33E-06	6.26E-05	Amino Acid

1-palmitoyl-2-oleoyl-GPE (16:0/18:1)	1.7799431	6.52E-06	6.32E-05	Lipid
1-palmitoyl-2-arachidonoyl-GPE (16:0/20:4)*	1.48724708	6.54E-06	6.32E-05	Lipid
sulfate of piperine metabolite C16H19NO3 (3)*	-3.0781476	6.93E-06	6.62E-05	Xenobiotics
1-stearoyl-2-arachidonoyl-GPC (18:0/20:4)	-0.518684	8.08E-06	7.64E-05	Lipid
S-methylcysteine sulfoxide	-2.9419451	8.75E-06	8.17E-05	Amino Acid
3-phenylpropionate (hydrocinnamate)	-3.6826924	9.68E-06	8.94E-05	Xenobiotics
hydroxypalmitoyl sphingomyelin (d18:1/16:0(OH))**	0.82608895	1.01E-05	9.27E-05	Lipid
eicosenedioate (C20:1-DC)*	-1.1921399	1.22E-05	0.00011014	Lipid
2-palmitoyl-GPC (16:0)*	-1.1948791	1.26E-05	0.00011286	Lipid
isoleucine	0.51303851	1.31E-05	0.00011556	Amino Acid
3-methylxanthine	-3.3420925	1.70E-05	0.00014857	Xenobiotics
1-arachidonoyl-GPC (20:4n6)*	-1.0048663	1.90E-05	0.00016438	Lipid
homostachydrine*	-4.2792315	2.08E-05	0.00017838	Xenobiotics
2-palmitoylglycerol (16:0)	2.91737945	2.12E-05	0.00017979	Lipid
alpha-hydroxyisovalerate	2.08996906	2.29E-05	0.0001922	Amino Acid
1-palmitoyl-2-oleoyl-GPC (16:0/18:1)	0.37268444	2.42E-05	0.00020144	Lipid
sphingomyelin (d18:2/24:2)*	-0.6309832	2.60E-05	0.00021441	Lipid
5-hydroxylysine	0.9255135	2.69E-05	0.00021924	Amino Acid
1-palmitoylglycerol (16:0)	3.84222523	2.97E-05	0.00023847	Lipid
3-methylhistidine	-1.2861219	2.99E-05	0.00023847	Amino Acid
epiandrosterone sulfate	-1.6718694	3.01E-05	0.00023847	Lipid
tryptophan	-0.6099883	3.28E-05	0.00025762	Amino Acid
sphingadienine	-2.9072649	3.82E-05	0.00029614	Lipid
3-hydroxyoctanoylcarnitine (2)	-1.1767537	3.84E-05	0.00029614	Lipid
N-palmitoyl-sphingosine (d18:1/16:0)	1.43592172	4.07E-05	0.00031065	Lipid
1-palmitoyl-2-linoleoyl-GPE (16:0/18:2)	2.37854667	4.10E-05	0.00031065	Lipid
2-linoleoylglycerol (18:2)	3.66785775	4.18E-05	0.00031357	Lipid
piperine	-4.9509192	4.56E-05	0.00033902	Xenobiotics
2-hydroxybutyrate/2-hydroxyisobutyrate	1.50839041	4.63E-05	0.00034075	Amino Acid
1-dihomo-linoleoylglycerol (20:2)	3.50966304	4.66E-05	0.00034075	Lipid
1-linoleoylglycerol (18:2)	4.76463647	4.91E-05	0.00035569	Lipid
palmitoyl-linoleoyl-glycerol (16:0/18:2) [2]*	1.86110471	4.95E-05	0.00035569	Lipid
hexanoylglutamine	2.20752979	5.37E-05	0.00038258	Lipid
eicosanedioate (C20-DC)	-0.9463843	5.80E-05	0.00040968	Lipid
dihomolinoleate (20:2n6)	1.14050475	6.08E-05	0.00042638	Lipid
2-oleoylglycerol (18:1)	4.64982054	6.17E-05	0.00042894	Lipid
dodecanedioate (C12)	-1.4164392	6.23E-05	0.00042931	Lipid
kynurenine	1.13625316	6.77E-05	0.00046286	Amino Acid
leucine	0.46689401	7.00E-05	0.00047323	Amino Acid
N-palmitoyl-sphingadienine (d18:2/16:0)*	0.98327491	7.03E-05	0.00047323	Lipid
10-undecenoate (11:1n1)	-1.0631228	7.41E-05	0.00049491	Lipid
trigonelline (N'-methylnicotinate)	-5.0617381	8.20E-05	0.00054297	Cofactors and Vitamins
sphinganine	0.77800574	8.32E-05	0.00054655	Lipid

oleoyl-linoleoyl-glycerol (18:1/18:2) [2]	0.96543199	8.85E-05	0.00057709	Lipid
phosphate	0.50450157	9.68E-05	0.00062619	Energy
palmitoyl-linoleoyl-glycerol (16:0/18:2) [1]*	1.85086109	9.80E-05	0.00062904	Lipid
theophylline	-2.7378518	0.00010088	0.00064274	Xenobiotics
6-hydroxyindole sulfate	-1.2168634	0.00010371	0.00065581	Xenobiotics
N-acetyl-aspartyl-glutamate (NAAG)	1.12508944	0.00011173	0.00070124	Amino Acid
tridecenedioate (C13:1-DC)*	-1.2478907	0.00011298	0.00070382	Lipid
ectoine	-2.145547	0.00011443	0.00070763	Xenobiotics
gamma-glutamylcitrulline*	-0.7871553	0.00012768	0.0007771	Peptide
lignoceroyl sphingomyelin (d18:1/24:0)	-0.82791	0.00012935	0.0007771	Lipid
1-stearoyl-2-arachidonoyl-GPE (18:0/20:4)	1.01594606	0.00012976	0.0007771	Lipid
glucuronide of piperine metabolite C17H21NO3 (5)*	-2.6423146	0.0001302	0.0007771	Xenobiotics
sphingomyelin (d18:2/16:0, d18:1/16:1)*	-0.3925137	0.00013029	0.0007771	Lipid
sphingomyelin (d17:2/16:0, d18:2/15:0)*	-0.7674504	0.00013273	0.00078609	Lipid
1-oleoylglycerol (18:1)	4.36508553	0.00013408	0.00078852	Lipid
1-palmitoleoyl-2-linolenoyl-GPC (16:1/18:3)*	-1.3769727	0.0001359	0.00079371	Lipid
2-aminobutyrate	0.74096608	0.00013785	0.00079703	Amino Acid
beta-cryptoxanthin	-1.4976624	0.00013837	0.00079703	Cofactors and Vitamins
linoleate (18:2n6)	1.16508107	0.00014006	0.0008013	Lipid
glucuronide of piperine metabolite C17H21NO3 (3)*	-2.9623229	0.00015833	0.0008997	Xenobiotics
1-arachidonylglycerol (20:4)	3.08084114	0.00016029	0.00090473	Lipid
N-palmitoyl-sphinganine (d18:0/16:0)	1.51249366	0.00016886	0.00094477	Lipid
tryptophan betaine	-2.5460441	0.00016963	0.00094477	Amino Acid
pyruvate	0.84546393	0.00017289	0.00095658	Carbohydrate
taurine	-0.8004011	0.00017718	0.0009718	Amino Acid
paraxanthine	-3.4784446	0.00017795	0.0009718	Xenobiotics
4-hydroxyphenylpyruvate	-1.9577417	0.00018943	0.0010278	Amino Acid
beta-hydroxyisovalerate	2.46825159	0.00021259	0.00114607	Amino Acid
pentose acid*	-5.0910869	0.00022835	0.00122322	Phase 2 Conjugates/Bilirubin Catabolites
pipecolate	1.74542849	0.00023773	0.0012654	Amino Acid
lactosyl-N-behenoyl-sphingosine (d18:1/22:0)*	-0.8162293	0.00025599	0.00135401	Lipid
glycerol	1.32483925	0.00025804	0.00135631	Lipid
thymol sulfate	-6.2202644	0.00031466	0.00164366	Xenobiotics
N-stearoyl-sphinganine (d18:0/18:0)*	2.32233536	0.00031826	0.00165221	Lipid
sphingomyelin (d18:2/23:1)*	-0.5643809	0.00033261	0.00171609	Lipid
glucuronide of piperine metabolite C17H21NO3 (4)*	-3.6298423	0.00033836	0.00172649	Xenobiotics
deoxycholate	-1.6325855	0.00033873	0.00172649	Lipid
retinol (vitamin A)	-0.9168255	0.00038106	0.00193057	Cofactors and Vitamins
N-stearoyl-sphingosine (d18:1/18:0)*	1.9979089	0.00038692	0.0019485	Lipid
2-hydroxystearate	-0.7339122	0.00039601	0.00198242	Lipid
1-arachidonoyl-GPE (20:4n6)*	0.93372106	0.0004216	0.0020863	Lipid
suberoylcarnitine (C8-DC)	1.51580494	0.00042172	0.0020863	Lipid
1-pentadecanoylglycerol (15:0)	3.18581143	0.00042511	0.00208879	Lipid

hexanoylcarnitine (C6)	1.10604074	0.00042758	0.00208879	Lipid
1-stearoyl-2-oleoyl-GPE (18:0/18:1)	1.47997325	0.00042968	0.00208879	Lipid
sphingomyelin (d18:1/18:1, d18:2/18:0)	-0.4396795	0.00044343	0.00214323	Lipid
ribitol	0.71841865	0.00046756	0.00224697	Carbohydrate
linoleoylcarnitine (C18:2)*	1.52601347	0.00047177	0.00225433	Lipid
palmitoyl-arachidonoyl-glycerol (16:0/20:4) [1]*	1.39640417	0.00048492	0.00230406	Lipid
glucose	0.44360673	0.00048808	0.00230603	Carbohydrate
beta-citrylglutamate	1.31183554	0.00049265	0.00231463	Amino Acid
S-1-pyrroline-5-carboxylate	-1.7004251	0.00054022	0.00252401	Amino Acid
sphingomyelin (d17:1/16:0, d18:1/15:0, d16:1/17:0)*	-0.4272961	0.0005649	0.00262477	Lipid
arachidoylcarnitine (C20)*	-0.7638189	0.00057846	0.00267302	Lipid
1-stearoyl-2-linoleoyl-GPE (18:0/18:2)*	1.58886243	0.00059459	0.00273251	Lipid
cortisone	-1.1920627	0.00060841	0.00278084	Lipid
oleoyl-linoleoyl-glycerol (18:1/18:2) [1]	0.81319689	0.00066301	0.00301399	Lipid
eicosenoate (20:1n9 or 1n11)	1.32521943	0.00067534	0.00305354	Lipid
docosatrienoate (22:3n3)	1.94028878	0.00074654	0.00335743	Lipid
oleate/vaccenate (18:1)	0.82238485	0.00075099	0.00335948	Lipid
N4-acetylcytidine	1.35813601	0.00077037	0.00342794	Nucleotide
3-hydroxyhippurate	-5.016849	0.00077501	0.00343043	Xenobiotics
cystine	0.72592965	0.00084016	0.00369935	Amino Acid
branched-chain, straight-chain, or cyclopropyl 10:1 fatty acid (1)*	-1.0726011	0.00087572	0.00383582	Phase 2 Conjugates/Bilirubin Catabolites
2-hydroxyglutarate	0.62223417	0.00089827	0.00391424	Lipid
N6,N6,N6-trimethyllysine	1.47189681	0.00097371	0.00422108	Amino Acid
1-oleoyl-2-linoleoyl-GPE (18:1/18:2)*	1.55603828	0.00103111	0.004447	Lipid
phenol sulfate	-1.3470137	0.00106784	0.00458189	Amino Acid
quinate	-7.7651542	0.00110508	0.00470804	Xenobiotics
linolenoylcarnitine (C18:3)*	-0.7617858	0.00110843	0.00470804	Lipid
3-formylindole	-0.5513449	0.00113537	0.00479824	Xenobiotics
gamma-glutamylhistidine	-0.6280182	0.00115661	0.00486353	Peptide
ceramide (d18:2/24:1, d18:1/24:2)*	0.70705308	0.00116908	0.0048915	Lipid
N-delta-acetylornithine	-1.7336193	0.00127433	0.00530552	Amino Acid
4-vinylphenol sulfate	-5.2014369	0.00128134	0.00530841	Xenobiotics
1-myristoylglycerol (14:0)	3.41044394	0.00129628	0.00534399	Lipid
hypotaurine	1.75987079	0.00135782	0.00554407	Amino Acid
N-acetyl-2-aminooctanoate*	-1.7212798	0.001358	0.00554407	Lipid
hypoxanthine	-1.7477892	0.00140839	0.00572199	Nucleotide
1-palmitoyl-2-docosahexaenoyl-GPE (16:0/22:6)*	1.69429234	0.00152658	0.00617236	Lipid
2-hydroxy-4-(methylthio)butanoic acid	2.08717737	0.00159274	0.00640905	Amino Acid
(N(1) + N(8))-acetylspermidine	1.9055226	0.00164144	0.00657359	Amino Acid
N1-methylinosine	1.38177416	0.00165355	0.00659069	Nucleotide
1,2,3-benzenetriol sulfate (2)	-4.5656637	0.00174728	0.00693142	Xenobiotics
3,4-dihydroxybutyrate	1.13284963	0.00176695	0.00697654	Lipid
formiminoglutamate	2.04785565	0.00179521	0.007055	Amino Acid

palmitate (16:0)	0.69540414	0.00187842	0.00733627	Lipid
perfluorohexanesulfonate (PFHxS)	-1.917288	0.00188707	0.00733627	Xenobiotics
4-methylcatechol sulfate	-1.2994912	0.00189295	0.00733627	Xenobiotics
glycochenodeoxycholate 3-sulfate	3.19393876	0.00204254	0.00787969	Lipid
pantothenate (Vitamin B5)	-0.9327916	0.00211595	0.00812563	Cofactors and Vitamins
3-indoxyl sulfate	-0.8902004	0.00213765	0.00817164	Amino Acid
phenylpyruvate	-0.9388062	0.00229131	0.00869363	Amino Acid
2-methylbutyrylcarnitine (C5)	1.93474678	0.00229487	0.00869363	Amino Acid
phenylalanine	0.41688061	0.00244761	0.00923069	Amino Acid
N-acetylisoleucine	0.84031776	0.00268728	0.01004841	Amino Acid
1,2-dipalmitoyl-GPE (16:0/16:0)*	1.25524554	0.00268834	0.01004841	Lipid
1-stearoyl-GPI (18:0)	-0.9960486	0.00270975	0.01008361	Lipid
betaine	-0.3983021	0.00280924	0.01040781	Amino Acid
glycosyl ceramide (d18:2/24:1, d18:1/24:2)*	-0.613	0.00284117	0.01047992	Lipid
lyxonate	-1.4298729	0.00289243	0.01060507	Carbohydrate
tetrahydrocortisone glucuronide (5)	-1.8603494	0.00290032	0.01060507	Lipid
androstenediol (3beta,17beta) monosulfate (2)	-0.9532706	0.0029144	0.01061045	Lipid
3-methyl catechol sulfate (1)	-6.6784791	0.00312067	0.01127715	Xenobiotics
picolinate	0.81753213	0.00312435	0.01127715	Amino Acid
taurochenodeoxycholic acid 3-sulfate	3.48006064	0.00315421	0.01133628	Lipid
sphingomyelin (d18:1/17:0, d17:1/18:0, d19:1/16:0)	-0.4167352	0.00321285	0.01140808	Lipid
ethyl alpha-glucopyranoside	-3.2571933	0.0032131	0.01140808	Xenobiotics

Table S4: Significantly altered metabolites in VV-ECMO subjects compared with healthy controls

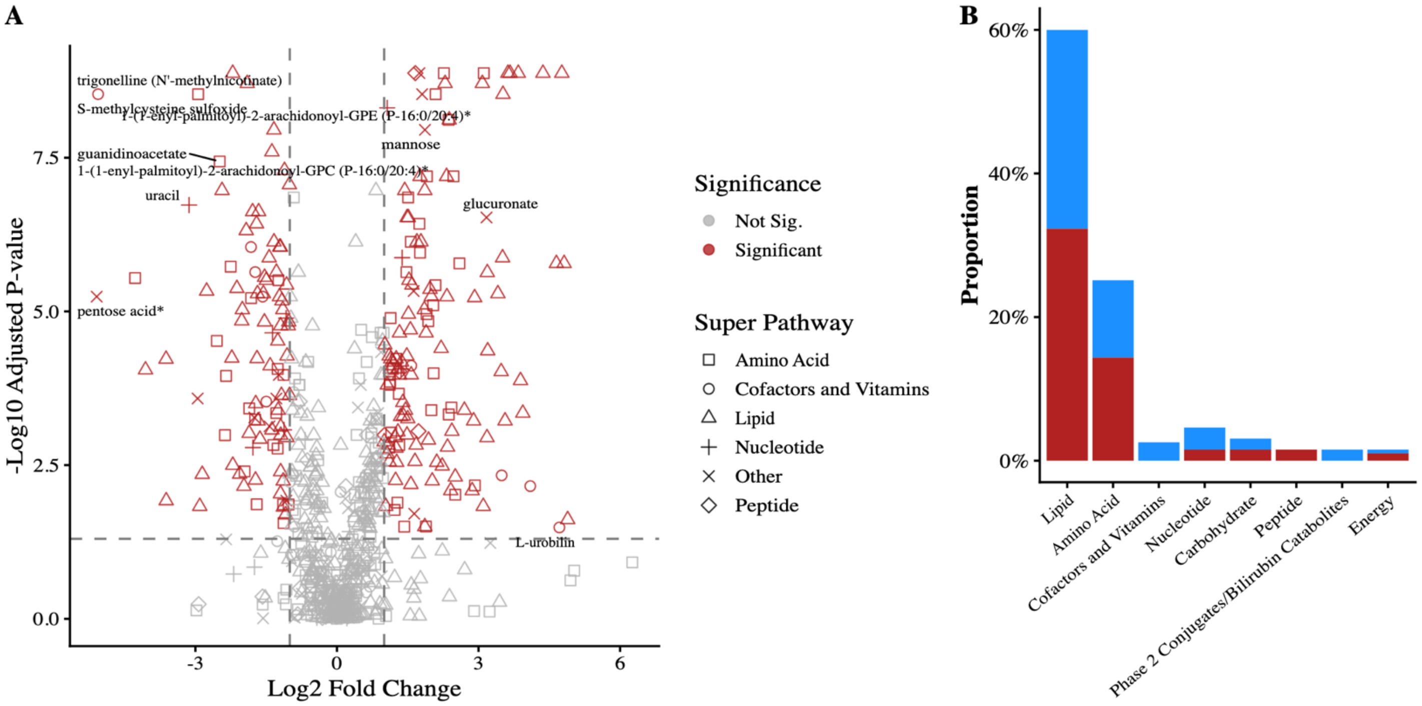

Figure S2. VV-ECMO vs healthy controls. Volcano plot depicting significantly altered metabolites (A). Bar plot depicting classes of altered metabolites (B).

Metabolite	log2fc	p.value	p.adj	SUPER_PATHWAY
**1-stearoyl-2-linoleoyl-GPI (18:0/18:2)**	**-1.4478136**	**5.71E-05**	**0.04334909**	**Lipid**
**1-palmitoyl-2-linoleoyl-GPC (16:0/18:2)**	**-0.4660377**	**0.000155**	**0.04334909**	**Lipid**
**1-palmitoyl-2-linoleoyl-GPE (16:0/18:2)**	**-1.377085**	**0.000155**	**0.04334909**	**Lipid**
1-palmitoyl-2-arachidonoyl-GPE (16:0/20:4)*	-1.05624	0.00024474	0.05133445	Lipid
1-oleoyl-2-linoleoyl-GPE (18:1/18:2)*	-1.4754817	0.00036711	0.06160134	Lipid
1-stearoyl-2-linoleoyl-GPE (18:0/18:2)*	-1.4360206	0.00053843	0.06433918	Lipid
sphingomyelin (d18:2/16:0, d18:1/16:1)*	-0.5611378	0.00053843	0.06433918	Lipid
1-linoleoyl-2-arachidonoyl-GPE (18:2/20:4)*	-2.1291498	0.00076686	0.06433918	Lipid
1-linoleoyl-GPI* (18:2)*	-1.1868103	0.00076686	0.06433918	Lipid
oleoyl-oleoyl-glycerol (18:1/18:1) [1]*	-1.3790348	0.00076686	0.06433918	Lipid
1-linoleoyl-GPG (18:2)*	-0.9971996	0.00107686	0.07529053	Lipid
hexadecadienoate (16:2n6)	-1.7200075	0.00107686	0.07529053	Lipid
1-palmitoyl-2-linoleoyl-GPI (16:0/18:2)	-1.2239053	0.0014766	0.08259143	Lipid
7-HOCA	1.42838688	0.0014766	0.08259143	Lipid
pyruvate	1.13663011	0.0014766	0.08259143	Carbohydrate
1-palmitoyl-2-docosahexaenoyl-GPE (16:0/22:6)*	-1.2196649	0.00200688	0.09354278	Lipid
2-pyrrolidinone	1.49413166	0.00200688	0.09354278	Amino Acid
cholesterol	-0.8952655	0.00200688	0.09354278	Lipid
1-palmitoleoylglycerol (16:1)*	-1.2963711	0.00267584	0.09354278	Lipid
1-stearoyl-2-linoleoyl-GPC (18:0/18:2)*	-0.4649112	0.00267584	0.09354278	Lipid
isoleucine	-0.8524722	0.00267584	0.09354278	Amino Acid
N-acetylputrescine	0.96787388	0.00267584	0.09354278	Amino Acid
N-stearoyl-sphingosine (d18:1/18:0)*	-1.4238149	0.00267584	0.09354278	Lipid
sphingomyelin (d18:1/17:0, d17:1/18:0, d19:1/16:0)	-0.6209834	0.00267584	0.09354278	Lipid
gamma-glutamylisoleucine*	-1.4491145	0.00353243	0.10158818	Peptide
leucine	-0.6400924	0.00353243	0.10158818	Amino Acid
N-palmitoyl-sphinganine (d18:0/16:0)	-0.907655	0.00353243	0.10158818	Lipid
N-stearoyl-sphinganine (d18:0/18:0)*	-1.5169395	0.00450637	0.10158818	Lipid
1-(1-enyl-palmitoyl)-2-arachidonoyl-GPC (P-16:0/20:4)*	0.72238727	0.00460113	0.10158818	Lipid
1-linoleoyl-GPE (18:2)*	-1.3553131	0.00460113	0.10158818	Lipid
1-oleoyl-2-arachidonoyl-GPE (18:1/20:4)*	-0.860977	0.00460113	0.10158818	Lipid
1-stearoyl-2-arachidonoyl-GPE (18:0/20:4)	-0.8665995	0.00460113	0.10158818	Lipid
alpha-tocopherol	-0.8775093	0.00460113	0.10158818	Cofactors and Vitamins
lactate	1.03671435	0.00460113	0.10158818	Carbohydrate
palmitoyl sphingomyelin (d18:1/16:0)	-0.2853935	0.00460113	0.10158818	Lipid
palmitoyl-linoleoyl-glycerol (16:0/18:2) [1]*	-1.0950179	0.00460113	0.10158818	Lipid
sphingomyelin (d18:1/24:1, d18:2/24:0)*	-0.7360839	0.00460113	0.10158818	Lipid
stearidonate (18:4n3)	-3.32284	0.00460113	0.10158818	Lipid
N-palmitoyl-sphingosine (d18:1/16:0)	-0.9272367	0.00593905	0.12153327	Lipid
palmitoyl-linoleoyl-glycerol (16:0/18:2) [2]*	-0.97017	0.00593905	0.12153327	Lipid
sphingomyelin (d18:1/20:2, d18:2/20:1, d16:1/22:2)*	-0.8987491	0.00593905	0.12153327	Lipid
behenoyl sphingomyelin (d18:1/22:0)*	-0.899445	0.00757066	0.12962822	Lipid

caprate (10:0)	-4.034336	0.00757066	0.12962822	Lipid
ceramide (d18:2/24:1, d18:1/24:2)*	-0.7083077	0.00757066	0.12962822	Lipid
gluconate	1.72906418	0.00757066	0.12962822	Xenobiotics
sphingomyelin (d18:1/18:1, d18:2/18:0)	-0.5099957	0.00757066	0.12962822	Lipid
sphingomyelin (d18:1/22:2, d18:2/22:1, d16:1/24:2)*	-0.6023588	0.00757066	0.12962822	Lipid
sphingomyelin (d18:2/18:1)*	-0.5953668	0.00757066	0.12962822	Lipid
sphingomyelin (d18:2/24:1, d18:1/24:2)*	-0.5466569	0.00757066	0.12962822	Lipid
N-palmitoyl-sphingadienine (d18:2/16:0)*	-1.1015689	0.00824627	0.13837243	Lipid
cortisone	1.44028411	0.00908784	0.14097461	Lipid
sphingomyelin (d17:2/16:0, d18:2/15:0)*	-0.874239	0.00914886	0.14097461	Lipid
1-stearoyl-2-docosahexaenoyl-GPE (18:0/22:6)*	-0.8681342	0.00957754	0.14097461	Lipid
1,2-dilinoleoyl-GPE (18:2/18:2)*	-1.3342086	0.00957754	0.14097461	Lipid
hexanoylglutamine	-1.5890652	0.00957754	0.14097461	Lipid
retinal	-0.9502769	0.00957754	0.14097461	Cofactors and Vitamins
sphingomyelin (d18:1/20:0, d16:1/22:0)*	-0.7081418	0.00957754	0.14097461	Lipid
saccharin	-4.4767882	0.00978531	0.14154958	Xenobiotics
2-palmitoleoyl-GPC (16:1)*	-1.1621477	0.01091705	0.14171201	Lipid
branched chain 14:0 dicarboxylic acid**	2.04522768	0.01091705	0.14171201	Lipid
galactonate	-4.2664869	0.01091705	0.14171201	Carbohydrate
sebacate (C10-DC)	-1.7042833	0.01109764	0.14171201	Lipid
1-palmitoyl-GPC (16:0)	-0.9685965	0.01199232	0.14171201	Lipid
biliverdin	1.84547004	0.01199232	0.14171201	Cofactors and Vitamins

Table S5. Significantly altered metabolites comparing all subjects with or without ABI

Metabolite	log2fc	p.value	p.adj	SUPER_PATHWAY
3-methoxycatechol sulfate (2)	-3.965308	0.0150785	0.51604938	Xenobiotics
pantoprazole	-8.2075575	0.0150785	0.51604938	Xenobiotics
1-methylurate	-3.0268925	0.01587302	0.51604938	Xenobiotics
1-palmitoyl-2-linoleoyl-GPC (16:0/18:2)	-0.4678249	0.01587302	0.51604938	Lipid
1-stearoyl-2-linoleoyl-GPC (18:0/18:2)*	-0.3577536	0.01587302	0.51604938	Lipid
1-stearoyl-2-linoleoyl-GPI (18:0/18:2)	-1.4402332	0.01587302	0.51604938	Lipid
1-stearoyl-GPI (18:0)	-0.9632171	0.01587302	0.51604938	Lipid
11beta-hydroxyandrosterone glucuronide	-1.5882893	0.01587302	0.51604938	Lipid
16-hydroxypalmitate	-1.5699791	0.01587302	0.51604938	Lipid
3beta,7alpha-dihydroxy-5-cholestenoate	1.3244356	0.01587302	0.51604938	Lipid
androsterone glucuronide	-3.6748261	0.01587302	0.51604938	Lipid
ceramide (d18:2/24:1, d18:1/24:2)*	-0.8654451	0.01587302	0.51604938	Lipid
cholesterol	-0.7017034	0.01587302	0.51604938	Lipid
cortolone glucuronide (1)	-1.7830597	0.01587302	0.51604938	Lipid
fructose	1.51321817	0.01587302	0.51604938	Carbohydrate
hexanoylglutamine	-3.6093095	0.01587302	0.51604938	Lipid
kynurenate	-3.4829595	0.01587302	0.51604938	Amino Acid
N-acetylputrescine	0.588555	0.01587302	0.51604938	Amino Acid
N-palmitoyl-sphingosine (d18:1/16:0)	-1.1215641	0.01587302	0.51604938	Lipid
N-stearoyl-sphingosine (d18:1/18:0)*	-1.3421393	0.01587302	0.51604938	Lipid
oleoyl ethanolamide	-1.2263809	0.01587302	0.51604938	Lipid
palmitoyl ethanolamide	-1.0409843	0.01587302	0.51604938	Lipid
4-methylguaiacol sulfate	-1.9965365	0.01945103	0.51604938	Xenobiotics
docosatrienoate (22:3n3)	-2.4164815	0.01945103	0.51604938	Lipid
(2 or 3)-decenoate (10:1n7 or n8)	-1.1358653	0.03174603	0.51604938	Lipid
1-linoleoyl-GPI* (18:2)*	-1.1277133	0.03174603	0.51604938	Lipid
1-oleoyl-GPI (18:1)	-0.7181242	0.03174603	0.51604938	Lipid
1-palmitoyl-2-linoleoyl-GPE (16:0/18:2)	-1.3270615	0.03174603	0.51604938	Lipid
1-palmitoyl-2-linoleoyl-GPI (16:0/18:2)	-1.3754021	0.03174603	0.51604938	Lipid
1-palmitoyl-2-oleoyl-GPC (16:0/18:1)	-0.3393904	0.03174603	0.51604938	Lipid
1-palmitoyl-2-palmitoleoyl-GPC (16:0/16:1)*	-0.7162367	0.03174603	0.51604938	Lipid
1-stearoyl-2-arachidonoyl-GPI (18:0/20:4)	-0.7376808	0.03174603	0.51604938	Lipid
17alpha-hydroxypregnanolone glucuronide	-1.3957171	0.03174603	0.51604938	Lipid
3-aminoisobutyrate	-1.7696134	0.03174603	0.51604938	Nucleotide
8-methoxykynurenate	-4.296771	0.03174603	0.51604938	Amino Acid
etiocholanolone glucuronide	-3.3229392	0.03174603	0.51604938	Lipid
gamma-glutamylisoleucine*	-1.5673024	0.03174603	0.51604938	Peptide
glutamine conjugate of C6H10O2 (1)*	-2.7235126	0.03174603	0.51604938	Phase 2 Conjugates/Bilirubin Catabolites
glutamine conjugate of C6H10O2 (2)*	-2.9679092	0.03174603	0.51604938	Phase 2 Conjugates/Bilirubin Catabolites
glutamine conjugate of C7H12O2*	-3.3703083	0.03174603	0.51604938	Phase 2 Conjugates/Bilirubin Catabolites
glycine conjugate of C10H14O2 (1)*	-1.1893996	0.03174603	0.51604938	Phase 2 Conjugates/Bilirubin Catabolites
hydroxy-N6,N6,N6-trimethyllysine*	-1.6046283	0.03174603	0.51604938	Amino Acid

laurate (12:0)	-2.1697383	0.03174603	0.51604938	Lipid
myo-inositol	-1.5616923	0.03174603	0.51604938	Lipid
myristoleate (14:1n5)	-2.1089353	0.03174603	0.51604938	Lipid
N2,N2-dimethylguanosine	-1.5867854	0.03174603	0.51604938	Nucleotide
octadecanedioate (C18)	-1.4572729	0.03174603	0.51604938	Lipid
palmitoleate (16:1n7)	-2.0405509	0.03174603	0.51604938	Lipid
tetradecadienoate (14:2)*	-1.5280507	0.03174603	0.51604938	Lipid
threonate	1.64425653	0.03174603	0.51604938	Cofactors and Vitamins
1,6-anhydroglucose	-5.4152397	0.0342039	0.51604938	Xenobiotics
isovalerylglycine	-2.5078616	0.0342039	0.51604938	Amino Acid
N-palmitoyl-sphingadienine (d18:2/16:0)*	-1.8694525	0.0342039	0.51604938	Lipid
N-stearoyl-sphinganine (d18:0/18:0)*	-1.4632278	0.03654634	0.51604938	Lipid

Table S6: Significantly altered metabolites comparing VA-ECMO subjects with or without ABI
